# Perceptions of firearms in a cohort of women exposed to intimate partner violence (IPV) in Central Pennsylvania

**DOI:** 10.1186/s12905-020-01134-y

**Published:** 2021-01-08

**Authors:** Laura Leuenberger, Erik Lehman, Jennifer McCall-Hosenfeld

**Affiliations:** 1 Department of Internal Medicine, University of Michigan, 338 Catherine St, Apt 1, Ann Arbor, MI 48104 USA; 2 Department of Public Health Sciences, Penn State University, Hershey, PA USA; 3 Department of Medicine, Penn State University, Hershey, PA USA

**Keywords:** Partner abuse, Abuse, Violence, Domestic violence, Gun violence, Battered women, Homicide

## Abstract

**Background:**

Almost one-half of U.S. women will experience intimate partner violence (IPV), defined as physical, sexual, or psychological harm by a current or former partner. IPV is associated with an increased risk of homicide, with firearms as the most commonly used weapon. We designed this study to better understand the correlation of interpersonal trauma exposures and demographic factors on firearm perceptions among a cohort of IPV-exposed women.

**Methods:**

Two hundred sixty-seven women in central Pennsylvania with exposure to IPV were surveyed about perceptions of gun access, safety, and gun presence in the home. Trauma variables included IPV type, IPV recency, unwanted sexual exposure, and adverse childhood experiences (ACEs). Multivariable analyses examined three questions examining firearm perceptions controlling for trauma exposures and demographics.

**Results:**

*Ease of firearm acquisition*: Women who were older (mean 44.92 years +/− SD 12.05), compared to women who were younger (40.91 +/− SD 11.81 years) were more likely to describe it as easy or very easy to acquire a gun (aOR 1.05, 95%CI 1.004, 1.10).

*Perceived safety in the proximity of a gun*: Women with the highest ACE score were less likely to feel safe with a gun nearby (aOR 0.31, 95%CI 0.14, 0.67).

*Odds of guns in the home*: Women who were divorced or separated (aOR 0.22, 95%CI 0.09, 0.54), women were widowed or single (aOR0.23, 95%CI 0.08, 0.67), and women who were partnered (aOR 0.45 95%CI 0.20, 0.97) had lower odds of having a gun in the home, compared to married women. There was no significant effect of the trauma variables on the odds of having a gun at home.

**Conclusions:**

Women with more severe childhood trauma felt less safe around firearms, but trauma exposures did not predict the perception of gun prevalence in the local community or gun ownership. Instead, demographic factors of marriage predicted presence of a gun in the home.

## Background

Nearly half (48%) of U.S. women experience intimate partner violence (IPV)—“physical violence, sexual violence, stalking and psychological aggression by a current or former intimate partner” [1]. One-quarter of women exposed to IPV sustain an injury from a partner, and IPV is the most common cause of nonfatal injury among women [2, 3]. Furthermore, women with a history of IPV are more likely to be homicide victims. Forty percent of femicides are perpetrated by an intimate partner, the majority with a firearm [4]. Research has consistently shown that the presence of guns increases the risk of a woman being murdered [4, 5], despite the political expediency of a popular narrative, promoted by the firearm industry, citing guns to be empowering for women’s self-defense [6].

In times of personal stress and natural disasters, intimate partner violence rates increase, as do rates of intimate partner homicide [7]. Most recently, the stress posed by the COVID-19 (novel coronavirus) pandemic, are anecdotally linked and temporally correlated with internationally increasing rates of intimate partner violence and homicide [8]. As the world attempts to slow the spread of this virus, movements to “Stay Home” are predicated on “home” being a safe place.

In the US context, gun prevalence in a community correlates to higher rates of IPV, although this association is confounded by regional and state variability. Rates of firearm-related IPV are highest in the states with highest firearm prevalence. Overall, there is a trend toward excess female mortality in states with high availability of firearms [9]. Many states have IPV-related firearm laws, most of which aim to prevent perpetrators of domestic violence from purchasing firearms; other states allow or require the removal of already owned firearms by police. States with laws removing guns from IPV perpetrators have lower rates of intimate partner related homicide [10]. These data are difficult to interpret however, given state level variability in both the laws themselves and their enforcement. For example, some states require removal of a perpetrator’s firearm only if the gun has been used to threaten the victim, while others require the abuser to be arrested [11]. Laws which prevent individuals who have a restraining order filed against them from owning or purchasing a firearm have been correlated with decrease in intimate partner homicide [12].

Despite the risks posed by firearms to IPV-exposed women, little is known about gun ownership and access in this population. Qualitative research on the topic has explored how women with a history of exposure to IPV feel, noting a diversity of opinions. Some women reported feeling danger when a partner had a gun, noting that the firearm could be a constant threat within the relationship. Other women perceived that a gun might protect them from an abuser [13, 14].

While there are demographics factors known to correlate to gun ownership, such as marriage and rurality [15], it is not known if these same factors are associated with proximity to a gun in high risk women, or if trauma exposures in these women affect their perceptions of guns. The National Gun Policy Survey of the National Opinion Research Center has shown that the possession of a firearm is strongly associated with living in a rural area as well as with being married [11, 16]. Furthermore, gun ownership appears to vary by race [11]. Age has been shown as a correlate of gun ownership, as Americans under 35 years old were less likely to own a gun than adults over 65 years old [11]. Increased household income correlated positively with ownership in the literature [11].

**Fig. 1:**
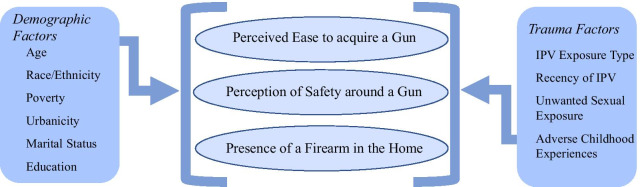
Conceptual Model of Factors Shaping IPV-Exposed Women’s Perceptions of Firearms

To understand if these correlates applied in a high-risk cohort of IPV-exposed women, we first reviewed the literature examining factors influencing firearm ownership and opinions about firearm safety to create a conceptual model for this study. Our conceptual model, created out of this literature review and shown in Fig. 1, characterizes gun ownership and opinions about firearm safety and access as being governed by two major categories: demographics and trauma exposures.

Given the increased mortality risk firearms pose to women who have a history of IPV, this study seeks to understand how this high-risk cohort perceives guns, and if the same factors that govern popular sentiment apply to this population. To help to understand these questions, we examined a cohort of IPV-exposed women with respect to perceptions of accessibility to firearms in their communities, perceptions of safety with a gun nearby, and the presence of firearms in the home. IPV victims’ perceptions were the focus of our analysis because victims are uniquely qualified to assess their own risk of lethality; that is, women who believe they are at increased risk of violence, are, in fact, at increased risk [5, 17]. We designed this study to contribute to an understanding of what factors are associated with perceptions of guns among victims of IPV and other interpersonal traumas, in hopes that clinicians and policymakers can help women to mitigate their risks.

## Methods

### Sample selection

The sample identification protocols for this study have been previously published [18]. The cohort was recruited between Fall 2013 and Spring 2014 in south central Pennsylvania, USA. Women were eligible for inclusion if they screened positive for a lifetime history of IPV based on the humiliation-afraid-rape-kick (HARK) screening instrument, a validated 4-item screen to identify IPV in healthcare settings [19], and left their contact information after completing the screening questionnaire (Additional file 1: Screener Survey). Participants subsequently completed the Baseline Survey (Additional file 2: Baseline Survey). Data collection for the 1 year follow up took place between Fall 2014 and Spring 2015 (Additional file 3: One Year Follow Up Survey).

Briefly, we identified a sample of 24,338 women ages 18-64 in south central Pennsylvania with least one primary care visit in the past year. A randomly selected subset of 2,500 women were invited to participate; surveys were received from 1,191 women from the clinical sample. The sample was stratified for rural residence using the zip-code based approximation of the Rural-Urban Commuting Area codes, a classification system based on city size and commuting practices [20]. Rural-residing women were oversampled to achieve appropriate numbers for analysis. To augment the cohort drawn from the healthcare setting, posters were also displayed at 26 domestic violence shelters in Central Pennsylvania, inviting women to participate in the survey online, by phone, or by mail. From this population, an additional 73 women were recruited to participate in response to these posters, yielding the final sample size of 1264 women who completed the screening survey.

Among this sample, those women who screened positive for lifetime exposure to IPV based on the humiliation-afraid-rape-kick (HARK) screening instrument, a validated 4-item screen to identify IPV in healthcare settings [19] and who left their contact information were contacted with an invitation to participate in a longitudinal study, requiring completion of a survey at baseline and one year later.

Of the women recruited via the ambulatory cohort, 500 women screened positive for IPV, and 270 participated in the baseline survey. From the participants recruited from the shelter sample, 60 women screened positive for IPV, and 38 participated in the baseline survey. These two subgroups were treated identically after initial recruitment. After 1 year, all women who completed the baseline survey were contacted and asked to complete the follow up survey. Among the women completing the follow up survey, 239 from the ambulatory cohort and 28 shelter participants (for a total 267 women) participated in the one-year follow-up survey. These 267 women form the analytic cohort for this analysis.

Study data were entered and managed within REDCap (Research Electronic Data Capture), a secure, web-based application designed to support data capture for research studies, hosted by Pennsylvania State University [21]. This study was conducted with approval from the Institutional Review Board (IRB) for all study protocol and study documents. All women reviewed a written or verbal informed consent and consented to participate in this research. To protect participants further, and due to the sensitive nature of this study, a Certificate of Confidentiality (CC-MH-12-204) was obtained from the National Institutes of Health for this research.

### Variables of interest

The follow up cohort of 267 women was assessed for the three primary outcomes surrounding firearms, of 1) perceptions of access (“How easy is it for people who live near you to get a gun?”), 2) perceptions of safety (“Does having a gun around make you feel safer or less safe?”), and 3) firearm proximity (“Are any firearms kept in or around your home?”) [16, 22]. As noted in Fig. 1, we hypothesized that trauma exposures would affect perceptions of firearm safety, in that women with a history of trauma would feel less safe around guns, be less likely to have guns in the home, and perceive guns to be readily available in their community.

Our primary independent variables were demographics and trauma exposures. To assess prior history of trauma, participants were screened for IPV recency (past-year vs. lifetime) and IPV type (physical vs nonphysical) using HARK [19]. Nonphysical IPV (humiliate-afraid) and physical IPV (rape-kick) were mutually exclusive categories, and participants were stratified into the physical IPV category if they had ever experienced physical IPV. The HARK question stem was modified to determine whether they had experienced IPV in their lifetimes compared to the past year. Additional interpersonal trauma exposures were unwanted sexual exposure [23] and adverse childhood experiences [24]. “Unwanted sexual exposure” was categorized as never, lifetime, or past-year [23]. “Adverse childhood experiences” (ACEs) were stratified by severity into tertiles. ACEs were determined using a definition taken from the ACE study (a collaborative research endeavor funded by the CDC and Kaiser Permanente [24].

To control for variation in the sample by demographics, we evaluated our cohort for age, marital status, urbanicity, poverty, education, and race/ethnicity. These variables were chosen because of their relevance to IPV, as well as their role in gun ownership trends. We considered whether our patients were near poverty (defined as 125% of the national poverty line) or not near poverty. Gun ownership also varies with region of the country, but our cohort is from within the same regional area, so we were unable to account for this variation.

### Data analysis

All variables were summarized with frequencies and percentages. Binomial or ordinal logistic regression, depending on the format of the outcome variable, was used to determine any unadjusted bivariate associations between each of the demographic and trauma exposure variables and each of the three firearm perception questions. Covariates were selected and retained for inclusion in the model based on their relationship to the outcomes variables as seen in the literature. As there were very few missing data, these were not included in analyses. We did not infer any missing data.

As noted above, significant data exists on the demographic variables associated with gun ownership, especially surrounding age, race/ethnicity, rurality, marital status, education, and income. Given that our outcomes variables included questions of guns in the home and also gun perceptions, we considered that these demographic variables were likely predictors of our outcome variables to be included in our analyses. To assess the relationship of gun ownership and perceptions with types of interpersonal trauma, we looked at different types of IPV, recency of IPV, unwanted sexual exposure, and ACEs to evaluate if these traumas were related to our outcomes variables. Interactions were not specifically tested in this model.

Multivariable analyses examined the associations of these exposure variables collectively with each of the three firearm perception questions while controlling for the demographic variables. All of the independent variables were tested for multicollinearity prior to inclusion in the model using variance inflation factor (VIF) statistics, and the fit of the multivariable models was assessed using the Pearson, Deviance, and Hosmer and Lemeshow goodness-of-fit tests. If the majority of these tests showed good model fit with *p* > 0.05, we accepted the model as having good fit, and this was the case for all three multivariable models. All analyses used a significance level of *p* < 0.05 and were performed using SAS version 9.4 [25].

## Results

As seen in Table 1, among the 266 respondents, the mean age was 44.48 years, with 89% identifying as white, non-Hispanic. Only 17% of this cohort were at or near poverty (income less than 150% the US poverty line). Of the cohort, 46% resided in urban areas, and 48% were married. Sixty-five percent experienced physical IPV; 21% reported IPV within the past year. For unwanted sexual exposure, 58% had been exposed in their lifetime. Almost one third of this cohort (32%) had experienced 4-10 Adverse Childhood Experiences. Bivariate data revealed significance in the relationship between marital status and the presence of a gun in the home. Also significant were the relationships between feeling unsafe around a firearm and the cohort with the highest number of Adverse Childhood Experiences.

**Table 1:** Trauma and demographic variables relating to firearm perceptions in a cohort of IPV-exposed woman

		Overall**	How easy is it for people who live near you to get a gun?			Are any firearms kept in or around your home?			Does having a gun around make you feel more or less safe?		
			Very Easy/ Easy	Hard/ Impossible		No	Yes		Very Safe/ Safe	Somewhat Less Safe/ Very Unsafe	
***Demographic variables***											
		*mean (SD)*	*mean (SD)*	*mean (SD)*	*p-value*	*mean (SD)*	*mean (SD)*	*p-value*	*mean (SD)*	*mean (SD)*	*p-value*
Age in years	*n* = 266	44.48 (12.13)	44.92 (12.05)	40.91 (11.81)	0.07	44.85 (12.19)	44.06 (12.18)	0.60	43.24 (12.34)	45.54 (11.86)	0.14
		*n (%)*	*n (%)*	*n (%)*	*p-value*	*n (%)*	*n (%)*	*p-value*	*n (%)*	*n (%)*	*p-value*
Race/ Ethnicity	Non-Hispanic white	236 (89%)	194 (86%)	31 (14%)	0.65	128 (55%)	106 (45%)	0.19	129 (58%)	92 (42%)	0.23
	non-white	28 (11%)	25 (89%)	3 (11%)		19 (68%)	9 (32%)		13 (46%)	15 (54%)	
Near poverty	Yes	42 (17%)	35 (87%)	5 (13%)	0.90	29 (69%)	13 (31%)	0.07	23 (55%)	19 (45%)	0.76
	No	210 (83%)	179 (88%)	24 (12%)		112 (54%)	96 (46%)		114 (57%)	85 (43%)	
Urban	Yes	121 (46%)	102 (88%)	14 (12%)	0.57	76 (63%)	45 (37%)	0.05*	60 (53%)	53 (47%)	0.23
	No	144 (54%)	118 (86%)	20 (14%)		72 (51%)	70 (49%)		83 (61%)	54 (39%)	
Marital status	Divorced/ Separated	47 (18%)	42 (89%)	5 (11%)	0.33	35 (74%)	12 (26%)	< 0.01*	22 (50%)	22 (50%)	0.48
	Married	127 (48%)	103 (85%)	18 (15%)		53 (42%)	73 (58%)		71 (60%)	47 (40%)	
	Partnered	60 (23%)	47 (82%)	10 (18%)		36 (61%)	23 (39%)		36 (60%)	24 (40%)	
	Widowed/ Single	32 (12%)	29 (97%)	1 (3%)		24 (75%)	8 (25%)		14 (48%)	15 (52%)	
Education	High school or less	62 (23%)	51 (85%)	9 (15%)	0.86	35 (56%)	27 (44%)	0.63	35 (58%)	25 (42%)	0.87
	Some College	84 (32%)	69 (87%)	10 (13%)		50 (60%)	33 (40%)		46 (58%)	33 (42%)	
	College Graduate	119 (45%)	101 (88%)	14 (12%)		63 (53%)	55 (47%)		61 (55%)	50 (45%)	
**Interpersonal trauma variables**											
Lifetime IPV exposure type	Non Physical (Humiliate-Afraid)	93 (35%)	77 (86%)	12 (14%)	0.94	51 (55%)	41 (45%)	0.85	48 (57%)	36 (43%)	0.93
	Physical (Rape-Kick)	174 (65%)	145 (87%)	22 (13%)		98 (57%)	75 (43%)		95 (57%)	73 (43%)	
Past year exposure to IPV	Yes	56 (21%)	45 (83%)	9 (17%)	0.41	29 (52%)	27 (48%)	0.45	24 (44%)	30 (56%)	0.04*
	No	211 (79%)	177 (88%)	25 (12%)		120 (57%)	89 (43%)		119 (60%)	79 (40%)	
Unwanted sexual exposure	Never	94 (37%)	77 (86%)	13 (14%)	0.77	46 (49%)	47 (51%)	0.11	59 (69%)	27 (31%)	0.01*
	Past Year	14 (5%)	12 (92%)	1 (8%)		11 (79%)	3 (21%)		4 (31%)	9 (69%)	
	Lifetime	149 (58%)	126 (88%)	18 (12%)		85 (57%)	63 (43%)		77 (53%)	68 (47%)	
Adverse childhood events	T1 (0-1)	96 (37%)	78 (83%)	15 (16%)	0.50	49 (51%)	47 (49%)	0.18	57 (66%)	29 (34%)	< 0.01*
	T2 (2-3)	78 (3%)	64 (88%)	9 (12%)		42 (55%)	34 (45%)		47 (64%)	27 (36%)	
	T3 (4-10)	82 (32%)	71 (90%)	8 (10%)		53 (65%)	29 (35%)		33 (41%)	48 (59%)	

* Significant using *p*-value of 0.05

**“Overall” percentages refer to those of the column (the entire study cohort); Specific variable column percents refer to the row

Multivariable analyses are shown in Table 2, noting both significant and not significant associations. Women who were older (aOR 1.05, 95% CI 1.004, 1.097) were more likely to report guns to be easy to acquire in their communities. Analysis of perceived safety in the proximity of a gun showed significance in the relationship of women with the highest ACE score, indicating a high level of childhood trauma, to be less likely to feel safe with a gun nearby (versus those with the lowest ACE score, aOR 0.31, 95% CI 0.36–1.58).

**Table 2:** Multivariate odds ratios of firearms perceptions among women with a history of exposure to intimate partner violence

	“How easy is it for people who live near you to get a gun?”^a^		“Are any firearms kept in or around your home?”^b^		“Does having a gun around make you feel safer or less safe?”^c^	
	aOR	95% CI	aOR	95% CI	aOR	95% CI
Age						
Mean	1.05	(1.00, 1.10)*	1.00	(0.97, 1.03)	0.99	(0.96, 1.02)
Race/ethnicity						
Non-white	Reference					
Non-Hispanic White	0.72	(0.17, 3.12)	1.29	(0.48, 3.43)	1.60	(0.63, 4.11)
Near poverty						
Not near poverty	Reference					
Near poverty	1.12	(0.28, 4.52)	0.99	(0.40, 2.43)	1.24	(0.52, 2.95)
Urban						
Urban	Reference					
Not urban	0.78	(0.31, 1.97)	0.66	(0.36, 1.22)	0.86	(0.46, 1.60)
Marital status						
Married	Reference					
Divorced/separated	1.66	(0.42, 6.56)	0.22	(0.09, 0.54)*	0.77	(0.34, 1.74)
Partnered	1.81	(0.58, 5.62)	0.45	(0.20, 0.97)*	0.93	(0.14, 2.08)
Widowed/single	6.27	(0.68, 58.12)	0.23	(0.08, 0.67)*	0.40	(0.14, 1.09)
Education						
College graduate	Reference					
High school or less	0.52	(0.15, 1.83)	0.94	(0.43, 2.08)	1.26	(0.56, 2.84)
Some college	0.82	(0.27, 2.43)	0.66	(0.32, 1.34)	1.38	(0.66, 2.88)
Lifetime IPV exposure type						
Non physical (humiliate-afraid)	Reference					
Physical (rape-kick)	1.61	(0.60, 4.33)	1.14	(0.58, 2.24)	1.68	(0.83, 3.40)
Past year exposure to IPV						
No	Reference					
Yes	0.45	(0.16, 1.23)	1.40	(0.68, 2.88)	0.73	(0.35, 1.50)
Unwanted sexual exposure						
Never	Reference					
Past year	1.47	(0.14, 15.97)	0.39	(0.08, 1.83)	0.31	(0.07, 1.31)
Lifetime	0.61	(0.22, 1.72)	0.96	(0.47, 1.93)	0.60	(0.29, 1.26)
Adverse childhood events						
0–1 events	Reference					
2–3 events	1.28	(0.46, 3.60)	0.77	(0.38, 1.55)	0.75	(0.36, 1.58)
4–10 events	2.72	(0.76, 9.71)	0.68	(0.32, 1.45)	0.31	(0.14, 0.67)*

^a^Higher aOR indicates “easier”

^b^Higher aOR indicates “Yes”

^c^Higher aOR indicates “safer”

*Indicates significant odds ratios

Multivariable analysis showed no significant association between any of the trauma exposure variables and having a gun at home. However, women who were divorced or separated (aOR 0.22, 95% CI 0.09–0.54), women who were partnered (aOR 0.45, 95% CI 0.20–0.97), and women who were widowed or single (aOR 0.23, 95% CI 0.08–0.67) had lower odds of having a gun in the home, compared to married women.

## Discussion

This study used quantitative analyses of survey data to explore how demographics and interpersonal traumas relate to IPV-exposed women’s perceptions of firearms. These analytic targets were chosen because women with a history of IPV are at high risk for violent injury. Our major findings were that a) older women perceived guns to be more accessible in their community, b) women with a high level of childhood trauma were less likely to feel safe with a gun nearby, and c) there was no association between trauma exposure and presence of a gun in the home.

Our finding that women with a high level of childhood trauma felt less safe near a gun, suggesting that a heightened perception of risk after trauma may extend from childhood to adulthood, was concordant with our hypothesis. Of note, firearms have never been shown to have a protective effect for women in violent intimate partner relationships [4]; indeed, the opposite is true. Despite this, 57% of our sample of IPV-exposed women felt having a gun around made them feel at least somewhat safer, this is similar to the 58% of American women (compared to 67% of American men) who think a gun makes them feel “safer” in 2015 [26, 27].

Regarding guns in the home, the various trauma exposure variables (IPV type, recency, unwanted sexual exposure and childhood adverse events) did not correlate with the presence of a gun in the home. Nationally, 30-40% of households in the US report having a firearm [15], so our cohort has a higher rate of gun ownership (44%) than the national average. Consistent with national trends, gun ownership was most correlated with demographic variables [15]. The high rate of firearms at home among IPV-exposed women may represent an area for intervention among professionals who encounter IPV-exposed women.

Together, our findings suggest that some trauma exposures likely impact how safe IPV-exposed women feel around guns, and yet are not reflected in whether or not they live in a home with a gun. Other influences govern proximity to a gun at home, which may prevent women from controlling their safety at home.

### Strengths and limitations of this study

Compared to other studies on this topic and in this population, a strength of this study is its size, and a sampling strategy that expanded the population of IPV victims from exclusively recruiting at shelters, to investigating the experiences of those seeking care from primary healthcare settings. Given the prevalence of IPV, this sampling method likely represents more diverse experiences among IPV-exposed women. Most other studies on this topic have recruited from only domestic violence shelters, which may represent a subset of women with a different pattern of violence than other IPV victims [28].

A weakness in this analysis is that the specific owner of the guns at home were unknown and could be the survey participant, a partner, or housemate; from our data, we were unable to determine if a current relationship reflected that of an abuser. While this study is larger than previous studies, it remains too small to effectively identify a large number of associations. Further study should be done to better characterize gun ownership in this population. We were also limited by our regional cohort, as gun opinions and ownership vary with geographic region of residence. Due to the limited racial and ethnic diversity in the cohort (although reflective of the larger population in the community sampled), we were unable to analyze by specific racial categories. Also limiting is our initial response rate from our participants recruited from the ambulatory care cohort, with an initial 2500 surveys resulting in 1191 responses (a response rate of 47.6%), concerning for potential for non-response bias; we are unable to know if non-responders varied in any significant way from responders. Unfortunately, we were unable to perform sensitivity analysis, given the lack of data on the non-responding group. This study only addresses female victims of IPV, while not addressing male victims; this focus is due to the increased risk of injury suffered by female as compared to male victims of IPV [29].

## Conclusions

This analysis may help to understand in the American context surrounding gun safety. The ultimate goal would be to inform policies which make women safer. Firearms are the most common form of weapon for intimate partner homicide in the U.S., but not in other high income countries [30]. International data from these high income countries, shows that overall female homicide and gun availability cluster together, with the U.S. being an extreme outlier in both [31]. An abusive partner’s access to a firearm in the home is associated with more severe IPV [30]. Understanding the risks that firearms pose in unsafe homes is increasingly important as the ongoing personal, political, and economic stresses wrought by the 2020 pandemic are unlikely to resolve in the near future.

As a public health practitioners, providers and policy makers must address the disconnect between having a gun in the home and the risks faced by IPV-exposed women. This study emphasizes the importance in empowering women to make decisions that make them safer and less likely victims of intimate partner violence and homicide. Furthermore, these findings are concordant with policies which remove guns from IPV perpetrators, which have been correlated to reduction in intimate partner homicide [10]. This study supports policies which help to educate women about their risks and to provide them with resources to make safe decisions as needed.

This novel study examined the perceptions of guns and risk by IPV-exposed women. Women with a trauma history are at an elevated risk for mortality from firearms, and can only be appropriately counselled if the risks are known. This data should inform public policy surrounding counseling women about intimate partner violence and gun ownership.

By understanding this, we hope to inform the debates surrounding intimate partner violence and gun ownership. Evidence suggests that limiting gun access of abusers decreases the number of intimate partner homicides [32]. Furthermore, it is important to understand the risks faced by these women, so they can be counselled appropriately to reduce these risks. The physician’s office is a place where women may seek help, and may provide an opportunity for intervention and prevention; IPV-exposed women have higher healthcare utilization than non IPV-exposed women [33, 34]. As such, strategies to engage women in their risks, and to understand their perspectives, would be valuable resources to decrease the risk of intimate partner violence, and ultimately intimate partner homicide.

## Supplementary Information

**Additional file 1:** Screener survey.

**Additional file 2:** Baseline survey.

**Additional file 3:** One year follow up survey.

## Data Availability

At the time data collection, the approving Institutional Review Board required that all datasets remain confidential and locally located, due to the sensitive nature of the data collected. Our IRB further explicitly states that our dataset will be destroyed after final publication of analyses. Thus, we are disallowed from creating a dataset that can be analyzed in perpetuity. If dataset is requested prior to study closure, it will be made available by the senior author (jmccallhosenfeld@pennstatehealth.psu.edu) on reasonable request.

## Abbreviations

ACE: Adverse Childhood Experiences
aOR: Adjusted Odds Ratio
CI: Confidence Interval
HARK: Humiliate Afraid Rape Kick
IRB: Institutional Review Board
IPV: Intimate Partner Violence
SD: Standard Deviation
