# Supplementary material for: Perceptions of firearms in a cohort of women exposed to intimate partner violence (IPV) in Central Pennsylvania
Source: BMC Womens Health. 2021 Jan 8;21:20. doi: 10.1186/s12905-020-01134-y (PMC7791958; doi:10.1186/s12905-020-01134-y)
Supplement: Supplementary file 1 — Additional file 1: Screener survey. [file 12905_2020_1134_MOESM1_ESM.pdf]

**Women's Health Screening Survey**

**YOUR WOMEN'S HEALTH SURVEY ID NUMBER:**

Please take a few minutes to complete this brief, confidential survey to help us understand women's health. Taking part in this survey is completely optional. If you choose not to complete this survey, it will not affect your health care or the services you receive in any way. This survey should take **less than five minutes to complete.**

There are three ways you can complete this survey. Please choose the one that works best for you.

1. To complete this survey online, please go to http://goo.gl/6qK2w and follow the directions on the website.
2. To complete the survey by mail, simply fill it out and return in the pre-paid envelope provided.
3. To complete the survey by telephone, please call the Women's Health Survey at our toll free number: 1-866-210-1137.

No matter how you complete the survey, your answers will be kept confidential. You may skip any questions you don't want to answer. Please review the enclosed Summary Explanation of Research and Consent for more information about how the researchers will protect your privacy. Thank you for helping with our research on women's health.

Based on your responses to this survey, you may be eligible to participate in our future research. If you participate in our next study (a 30 minute survey), you will receive a $25 gift card. **If you wish to participate in our next study and receive a $25 gift card, you must leave your contact information at the end of this survey.**

The first few questions will help us to understand your health and health risks.

**1.** In general, how would you describe your own health? (check one answer)

- ☐ Excellent
- ☐ Very good
- ☐ Good
- ☐ Fair
- ☐ Poor

**2.** In the past 5 years, has a doctor or other health professional told you that you have any of the following health problems or conditions?

| | | | |
|---|---|---|---|
| **2a**. Hypertension or high blood pressure | ☐ Yes | ☐ No | ☐ Don't know |
| **2b**. Heart attack or any other heart disease | ☐ Yes | ☐ No | ☐ Don't know |
| **2c**. Cancer | ☐ Yes | ☐ No | ☐ Don't know |
| **2d**. Diabetes | ☐ Yes | ☐ No | ☐ Don't know |
| **2e**. Anxiety or depression | ☐ Yes | ☐ No | ☐ Don't know |
| **2f**. Osteoporosis or brittle bones | ☐ Yes | ☐ No | ☐ Don't know |
| **2g**. Arthritis | ☐ Yes | ☐ No | ☐ Don't know |
| **2h**. Obesity | ☐ Yes | ☐ No | ☐ Don't know |
| **2i**. Asthma | ☐ Yes | ☐ No | ☐ Don't know |
| **2g**. Other: ______________________________ | ☐ Yes | ☐ No | ☐ Don't know |

**3.** Do you now smoke cigarettes every day, some days or not at all?
- ☐ Every day
- ☐ Some days
- ☐ Not at all

**4.** During the **past month**, other than your regular job, did you participate in any physical activities at least three times per week? Physical activity includes but is not limited to activities such as running, calisthenics, or walking for exercise.
- ☐ Yes
- ☐ No

**5.** How many times have you visited a doctor or other health care provider **in the last 12 months**, not counting overnight stays in a hospital? Number of visits: ________________

**6.** In the **past 12 months**, how many times have you used alcohol: (check one answer)
- ☐ Never
- ☐ Once or Twice
- ☐ Monthly
- ☐ Weekly
- ☐ Daily or Almost Daily

**7.** In the **past 12 months**, how many times have you used tobacco products: (check one answer)
- ☐ Never
- ☐ Once or Twice
- ☐ Monthly
- ☐ Weekly
- ☐ Daily or Almost Daily

**8.** In the **past 12 months**, how many times have you used prescription drugs for reasons other than for medical use (i.e., when you weren’t sick): (check one)

- ☐ Never
- ☐ Once or Twice
- ☐ Monthly
- ☐ Weekly
- ☐ Daily or Almost Daily

**9.** In the **past 12 months**, how many times have you used any illegal drugs: (check one)

- ☐ Never
- ☐ Once or Twice
- ☐ Monthly
- ☐ Weekly
- ☐ Daily or Almost Daily

**Some women struggle with drug, alcohol or other mental health issues. If you would like more information about these issues call the National Helpline at 800-662-HELP (4357) or visit the online treatment locator at http://www.samhsa.gov/treatment.**

**10.** Relationship stress with your current or former partners (i.e., boyfriends or girlfriends) can affect your health. In the **past 12 months** do any of the following apply to you? In the past 12 months, I was…

| | | | |
|---|---|---|---|
| **10a**. Humiliated or emotionally abused in other ways by a partner or ex-partner | ☐ Yes | ☐ No | ☐ Don't know |
| **10b**. Afraid of a partner or ex-partner | ☐ Yes | ☐ No | ☐ Don't know |
| **10c**. Raped or forced to have any kind of sexual activity by a partner or ex-partner | ☐ Yes | ☐ No | ☐ Don't know |
| **10d**. Kicked, hit, slapped, or otherwise physically hurt by a partner or ex-partner | ☐ Yes | ☐ No | ☐ Don't know |

**11.** Thinking back a little more, in **your lifetime** do any of the following apply to you? In my lifetime, I was…

| | | | |
|---|---|---|---|
| **11a**. Humiliated or emotionally abused in other ways by a partner or ex-partner | ☐ Yes | ☐ No | ☐ Don't know |
| **11b**. Afraid of a partner or ex-partner | ☐ Yes | ☐ No | ☐ Don't know |
| **11c**. Raped or forced to have any kind of sexual activity by a partner or ex-partner | ☐ Yes | ☐ No | ☐ Don't know |
| **11d**. Kicked, hit, slapped, or otherwise physically hurt by a partner or ex-partner | ☐ Yes | ☐ No | ☐ Don't know |

**If you need to talk about domestic violence or abuse feel free to call this toll-free and confidential hotline 1-800-799-SAFE (7233). Call 911 or your local emergency number if you are in immediate danger. Information can also be found at: http://www.thehotline.org.**

The next questions will help us to understand who you are.

**12.** What is your age? _______________

**13.** What is the zip code where you live? ___________

**14.** How would you best describe your race/ethnicity? (check all that apply)

- ☐ White
- ☐ African American/Black
- ☐ Asian
- ☐ Native American/American Indian
- ☐ Native Hawaiian/Pacific Islander/Alaskan Native
- ☐ Other: ___________________________________

**15.** Would you describe yourself as Hispanic?

- ☐ Yes
- ☐ No

**16.** What is the last grade or class you completed in school? (check one)
- ☐ Less than high school
- ☐ High school graduate (grade 12 or GED certificate)
- ☐ Some college after high school (no four-year degree)
- ☐ College graduate or greater (BS, BA, or other four-year degree)

**17.** What is your current relationship status? (check one)
- ☐ Married
- ☐ Living with a partner
- ☐ Partnered, but not living together
- ☐ Widowed
- ☐ Divorced
- ☐ Separated
- ☐ Not in a relationship

**18.** What is the gender of your current or most recent partner?
- ☐ Male
- ☐ Female
- ☐ Other: ________________________________

**19.** Last year, in 2012, what was your total household income from all sources before taxes? (check one)
- ☐ Under $25,000
- ☐ $25,000 – $49,999
- ☐ $50,000 - $99,999
- ☐ $100,000+
- ☐ Don't know

**20.** How would you best describe your health insurance?
- ☐ Private health insurance
- ☐ Public health insurance (Medicaid, Medicare)
- ☐ Uninsured
- ☐ Other: ________________________________

**21.** How did you hear about the Women's Health Study?
- ☐ Letter
- ☐ Poster
- ☐ Other: ______________________________

---

Thank you for completing our women's health survey. Please take a minute to look at the next page, which includes useful information about women's health.

**We would like to contact you again for future studies of women's health. Our next study is a 30-minute survey. If you are eligible and decide to participate in the next study, you will receive a $25 gift card. Please provide your contact information below if you are interested in participating in future studies.**

**Your confidentiality and safety are important to us. When contacting you, we will always say that we are calling from the "women's health study."**

If I am eligible, I would like to be contacted to participate in a future survey: *(please check one)*

- ☐ Yes
- ☐ No

Name: ____________________________________________________

Private Number (1):___________________________________________

Private Number (2):___________________________________________

Private Number (3):___________________________________________

Best Time to Contact You: ______________________________________

May we leave a message? ______________________________________

Email Address (if you prefer email): ______________________________

------------------------------------------TEAR OFF PORTION-----------------------------------------------

*Save this information in case you want to contact us!*
*Women's Health Study*
*Email: whs@hmc.psu.edu*
*Toll Free Phone: 1-866-210-1137*
