# Supplementary material for: Perceptions of firearms in a cohort of women exposed to intimate partner violence (IPV) in Central Pennsylvania
Source: BMC Womens Health. 2021 Jan 8;21:20. doi: 10.1186/s12905-020-01134-y (PMC7791958; doi:10.1186/s12905-020-01134-y)
Supplement: Supplementary file 2 — Additional file 2: Baseline survey. [file 12905_2020_1134_MOESM2_ESM.pdf]

Women's Health Survey

Thank you for taking the time to complete this survey for the Women's Health Study at Penn State.

This survey will take approximately 30 minutes.

Remember, in this women's health survey, we ask some questions that can be personal. We ask that you are in a safe and private location and that you are confident that nobody can see your responses if you do not want them to. You may skip any questions that you would prefer not to answer. You may stop the survey, or save and resume, at any time. There are no correct or incorrect responses.

Please review the Consent Summary before proceeding. Your completion of the survey implies your voluntary consent to participate in this research.

[Attachment: "Summary Explanation of Research and Consent for Survey.pdf"]

Do you agree to participate in this research as explained in the Research Consent Form?

- ☐ Yes
- ☐ No

Please enter your Participant ID Number: ______________________________

Please click the "now" button to the right, and begin the survey. ______________________________

A1. The first few questions will help us understand your overall health status.

1	In general, would you say your health is:	☐ Excellent<br>☐ Very Good<br>☐ Good<br>☐ Fair<br>☐ Poor
2	Do you take any prescription drugs on a regular basis?	☐ Yes<br>☐ No
3	Do you take any prescription medications specifically for depression, anxiety, or other emotional problem?	☐ Yes<br>☐ No
	Please include name of drug	______________________________
4	Have you been pregnant in the last year?	☐ Yes<br>☐ No

A2. Now thinking about any medical tests you may have had in the last 2 YEARS, have you had a...

Blood cholesterol test
☐ Yes
☐ No

Physical or check-up exam by a doctor or health care provider
☐ Yes
☐ No

Mammogram
☐ Yes
☐ No

Pap smear or pap test
☐ Yes
☐ No

Colon cancer screening
☐ Yes
☐ No

Test for HIV, the virus that causes AIDS
☐ Yes
☐ No

Test for any other sexually transmitted disease besides HIV/AIDS
☐ Yes
☐ No

Blood pressure check
☐ Yes
☐ No

A3. In the past 5 YEARS, has a doctor or other health professional told you that you have any of the following health problems or conditions?

Hypertension or high blood pressure
☐ Yes
☐ No

High cholesterol
☐ Yes
☐ No

Heart attack or any other heart disease
☐ Yes
☐ No

Cancer
☐ Yes
☐ No

Diabetes
☐ Yes
☐ No

Depression
☐ Yes
☐ No

Anxiety
☐ Yes
☐ No

Post-Traumatic Stress Disorder (PTSD)
☐ Yes
☐ No

Osteoporosis or brittle bones
☐ Yes
☐ No

Arthritis
☐ Yes
☐ No

Asthma
☐ Yes
☐ No

Overweight
☐ Yes
☐ No

Obesity
☐ Yes
☐ No

Anemia or other blood disorder
☐ Yes
☐ No

Other? Please describe: ______________________________

A4. This section will ask about your activity level.

1 During the PAST MONTH, other than your regular job, did you participate in any physical activities such as working out, running, brisk walking, tennis, swimming, or golf?

☐ Yes
☐ No

2 How many MINUTES per week do you usually get moderate-intensity exercise such as brisk walking ballroom dancing, or gardening? Please estimate.

__________________________________ (minutes)

3 How many MINUTES per week do you usually get vigorous-intensity exercise such as jogging, aerobic dancing, or jumping rope?

__________________________________ (minutes)

A5. In the PAST YEAR, how many times have you used the following?

4 or more drinks of alcohol in one day

- ☐ Never
- ☐ Once or Twice
- ☐ Monthly
- ☐ Weekly
- ☐ Daily or almost daily

Tobacco products, such as cigarettes, cigars, or chewing tobacco

- ☐ Never
- ☐ Once or Twice
- ☐ Monthly
- ☐ Weekly
- ☐ Daily or almost daily

Prescription drugs for NONmedical reasons (for example: taking a pain medication for relaxation instead of pain relief)

- ☐ Never
- ☐ Once or Twice
- ☐ Monthly
- ☐ Weekly
- ☐ Daily or almost daily

Illegal drugs

- ☐ Never
- ☐ Once or Twice
- ☐ Monthly
- ☐ Weekly
- ☐ Daily or almost daily

Some women struggle with drug or alcohol issues. If you would like more information about these issues call the National Helpline at 800-622-HELP (4357) or visit the online treatment locator at http://www.samhsa.gov/treatment.

A6. This section will ask about your eating habits.

1 Are you satisfied with your eating patterns?
☐ Yes
☐ No

2 Do you ever eat in secret?
☐ Yes
☐ No

3 Does your weight affect the way you feel about yourself?
☐ Yes
☐ No

4 Have any members of your family suffered with an eating disorder?
☐ Yes
☐ No

5 Do you currently suffer with or have you ever suffered in the past with an eating disorder?
☐ Yes
☐ No

If you are concerned that you or someone you care about is affected by an eating disorder, you may find out more information on the National Eating Disorders Association website: http://www.nationaleatingdisorders.org/. A toll free, confidential helpline is available Monday-Friday, 9:00 am- 5:00 pm, Eastern Standard Time: 1-800-931-2237.

B. In the next section, we are interested in understanding more about your mood and feelings.

1 Over the last TWO WEEKS, how often have you felt any of the following?

I feel sad, down in the dumps, or unhappy
- ☐ Not at all
- ☐ Rarely
- ☐ Sometimes
- ☐ Often
- ☐ Most of the time

I can't concentrate or focus
- ☐ Not at all
- ☐ Rarely
- ☐ Sometimes
- ☐ Often
- ☐ Most of the time

Nothing seems to give me much pleasure
- ☐ Not at all
- ☐ Rarely
- ☐ Sometimes
- ☐ Often
- ☐ Most of the time

I feel tired, I have no energy
- ☐ Not at all
- ☐ Rarely
- ☐ Sometimes
- ☐ Often
- ☐ Most of the time

I have had thoughts of suicide
- ☐ Not at all
- ☐ Rarely
- ☐ Sometimes
- ☐ Often
- ☐ Most of the time

If you are thinking of harming yourself, please call 911, your local emergency number, or the National Suicide Prevention Hotline at: 800-273-TALK (8255).

Please check here to indicate that you understand that you should seek help if you are feeling suicidal.
- ☐ I understand

2 Over the last TWO WEEKS, how often have you felt any of the following?

I have difficulty sleeping
- ☐ Not at all
- ☐ Rarely
- ☐ Sometimes
- ☐ Often
- ☐ Most of the time

I have been sleeping too much
- ☐ Not at all
- ☐ Rarely
- ☐ Somtimes
- ☐ Often
- ☐ Most of the time

I have lost my appetite
- ☐ Not at all
- ☐ Rarely
- ☐ Sometimes
- ☐ Often
- ☐ Most of the time

I have been eating more
- ☐ Not at all
- ☐ Rarely
- ☐ Sometimes
- ☐ Often
- ☐ Most of the time

I feel tense, anxious, or can't sit still
- ☐ Not at all
- ☐ Rarely
- ☐ Sometimes
- ☐ Often
- ☐ Most of the time

I feel worried or fearful
- ☐ Not at all
- ☐ Rarely
- ☐ Sometimes
- ☐ Often
- ☐ Most of the time

I have attacks of anxiety or panic
- ☐ Not at all
- ☐ Rarely
- ☐ Sometimes
- ☐ Often
- ☐ Most of the time

I worry about dying or losing control
- ☐ Not at all
- ☐ Rarely
- ☐ Sometimes
- ☐ Often
- ☐ Most of the time

I am nervous or shaky in social situations
- ☐ Not at all
- ☐ Rarely
- ☐ Sometimes
- ☐ Often
- ☐ Most of the time

I have nightmares or flashbacks
- ☐ Not at all
- ☐ Rarely
- ☐ Sometimes
- ☐ Often
- ☐ Most of th time

I am jumpy or feel startled easily
- ☐ Not at all
- ☐ Rarely
- ☐ Sometimes
- ☐ Often
- ☐ Most of the time

I avoid places that strongly remind me of a bad experience
- ☐ Not at all
- ☐ Rarely
- ☐ Sometimes
- ☐ Often
- ☐ Most of the time

I feel dull, numb, or detached
- ☐ Not at all
- ☐ Rarely
- ☐ Sometimes
- ☐ Often
- ☐ Most of the time

I can't get certain thoughts out of my mind
- ☐ Not at all
- ☐ Rarely
- ☐ Sometimes
- ☐ Often
- ☐ Most of the time

I feel I must repeat certain acts or rituals
- ☐ Not at all
- ☐ Rarely
- ☐ Sometimes
- ☐ Often
- ☐ Most of the time

I feel the need to check and recheck things
- ☐ Not at all
- ☐ Rarely
- ☐ Sometimes
- ☐ Often
- ☐ Most of the time

3a AT ANY TIME IN YOUR LIFE have you:

Had more energy than usual
- ☐ Not at all
- ☐ Rarely
- ☐ Sometimes
- ☐ Often
- ☐ Most of the time

Felt unusually irritable or angry
- ☐ Not at all
- ☐ Rarely
- ☐ Sometimes
- ☐ Often
- ☐ Most of the time

Felt unusually excited, revved up, or high
- ☐ Not at all
- ☐ Rarely
- ☐ Sometimes
- ☐ Often
- ☐ Most of the time

Needed less sleep than usual
- ☐ Not at all
- ☐ Rarely
- ☐ Sometimes
- ☐ Often
- ☐ Most of the time

3b Please indicate whether any of the above symptoms:

Interfere with work or school
- ☐ Not at all
- ☐ Rarely
- ☐ Sometimes
- ☐ Often
- ☐ Most of the time

Affect my relationships with friends or family
- ☐ Not at all
- ☐ Rarely
- ☐ Sometimes
- ☐ Often
- ☐ Most of the time

Have led to my using alcohol to get by
- ☐ Not at all
- ☐ Rarely
- ☐ Sometimes
- ☐ Often
- ☐ Most of the time

Have led to my using illegal drugs
- ☐ Not at all
- ☐ Rarely
- ☐ Sometimes
- ☐ Often
- ☐ Most of the time

Some women struggle with drug, alcohol or other mental health issues. If you would like more information about these issues call the National Helpline at 800-662-HELP (4357) or visit the online treatment locator at http://www.samhsa.gov/treatment.

4 Are you receiving treatment from a doctor or other health professional for any type of mental health condition or emotional problem?
- ☐ Yes
- ☐ No

C1. The next section will ask you questions about your interpersonal relationships.

1 Have you been involved in a relationship with a boyfriend, girlfriend, husband, wife, or other partner in the PAST YEAR?

☐ Yes
☐ No

2 Do you consider yourself to be:

☐ Heterosexual or straight
☐ Gay or lesbian
☐ Bisexual
☐ Other

Other: ______________________________

3 In the PAST YEAR, who have you had sex with?

☐ Men only
☐ Women only
☐ Both Men and Women
☐ I did not have sex in the past year
☐ Other

Other: ______________________________

C2. Relationship stress and violence are common in many people's lives and can affect women's health.

1 In your ADULT LIFETIME (since age 18), have you been humiliated or emotionally abused in other ways by your partner or ex-partner?
- ☐ Yes
- ☐ No

1a Has this happened within the PAST YEAR?
- ☐ Yes
- ☐ No

Has this become more frequent over the PAST YEAR?
- ☐ Yes
- ☐ No

Has this become more severe over the PAST YEAR?
- ☐ Yes
- ☐ No

1b How old were you the first time you were humiliated or emotionally abused by your partner or ex-partner? ______________________

1c How old were you the most recent time you were humiliated or emotionally abused by a partner or ex-partner? ______________________

1d What is the total number of partners or ex-partners who have humiliated or emotionally abused you since age 18? ______________________

2 In your ADULT LIFETIME (since age 18), have you been afraid of your partner or ex-partner?
- ☐ Yes
- ☐ No

2a Within the LAST YEAR, have you been afraid of your partner or your ex-partner?
- ☐ Yes
- ☐ No

Has this become more frequent over the PAST YEAR?
- ☐ Yes
- ☐ No

Has this become more severe over the PAST YEAR?
- ☐ Yes
- ☐ No

2b How old were you the first time you were afraid of your partner or ex-partner? ______________________

2c How old were you the most recent time you were afraid of your partner or ex-partner? ______________________

2d What is the total number of partners or ex-partners you have been afraid of since age 18? ______________________

3 In your ADULT LIFETIME (since age 18), have you been raped or forced to have any kind of sexual activity by your partner or ex-partner?
- ☐ Yes
- ☐ No

3a Has this happened within the PAST YEAR?
- ☐ Yes
- ☐ No

Has this become more frequent over the PAST YEAR?
- ☐ Yes
- ☐ No

Has this become more severe over the PAST YEAR?
- ☐ Yes
- ☐ No

3b How old were you the first time you were raped or forced to have any kind of sexual activity by your partner or ex-partner? ______________________

3c How old were you the most recent time you were raped or forced to have any kind of sexual activity by your partner or ex-partner? ________________________________

3d What is the total number or partners or ex-partners who have raped or forced you to have any kind of sexual activity since age 18? ________________________________

4 In your ADULT LIFETIME (since age 18), have you been kicked, hit, slapped, or otherwise physically hurt by your partner or ex-partner?
- ☐ Yes
- ☐ No

4a Has this happened within the PAST YEAR?
- ☐ Yes
- ☐ No

Has this become more frequent over the PAST YEAR?
- ☐ Yes
- ☐ No

Has this become more severe over the PAST YEAR?
- ☐ Yes
- ☐ No

4b How old were you the first time you were kicked, hit, slapped, or otherwise physically hurt by your partner or ex-partners? ________________________________

4c How old were you the most recent time you were kicked, hit, slapped, or otherwise physically hurt by your partner or ex-partners? ________________________________

4d What is the total number of partners or ex-partners who have kicked, hit, slapped, or otherwise physically hurt you since age 18? ________________________________

If you need to talk about domestic violence or abuse feel free to call this toll-free and confidential hotline 1-800-799-SAFE (7233).

If you need to talk to someone regarding rape or sexual violence please contact the National Sexual Assault Hotline at 1-800-656-HOPE (4673), a free and confidential service. Information call also be found at the Rape, Abuse & Incest National Network website: http://www.rainn.org/.

Call 911 or your local emergency number if you are in immediate danger. Information can also be found at: http://www.thehotline.org.

C3. Women who experience problems in their relationships have many ways of coping or seeking help.

1 In the PAST YEAR, have you done any of the following because of a problem with your partner or ex-partner?

I tried to get help from a member of the clergy	☐ Yes<br>☐ No
I tried to get help from an employer or coworker	☐ Yes<br>☐ No
I talked to a doctor or nurse about abuse or relationship trouble	☐ Yes<br>☐ No
I called a mental health counselor for myself	☐ Yes<br>☐ No
I tried to get my partner into counseling	☐ Yes<br>☐ No
I stayed in a shelter	☐ Yes<br>☐ No
I talked to someone at a domestic violence program, shelter or hotline	☐ Yes<br>☐ No
I tried to get help for alcohol or substance abuse	☐ Yes<br>☐ No
I tried to get my partner help for alcohol or substance abuse	☐ Yes<br>☐ No
I filed for a protection order	☐ Yes<br>☐ No
I filed or tried to file criminal charges	☐ Yes<br>☐ No
I sought help from legal aid	☐ Yes<br>☐ No
I called the police	☐ Yes<br>☐ No
I hid the car or house keys	☐ Yes<br>☐ No
I kept money and other valuables hidden	☐ Yes<br>☐ No
I developed a code so others would know I was in danger	☐ Yes<br>☐ No
I worked out an escape plan	☐ Yes<br>☐ No
I removed or hid weapons	☐ Yes<br>☐ No
I kept important phone numbers I could use to get help	☐ Yes<br>☐ No
I kept an extra supply of basic necessities for myself or my children	☐ Yes<br>☐ No

I hid important papers from my partner
☐ Yes
☐ No

I put a knife, gun, or other weapon where I could get it
☐ Yes
☐ No

I changed the locks or somehow improved security
☐ Yes
☐ No

I talked to family or friends about what to do to protect me or my children
☐ Yes
☐ No

I stayed with family or friends
☐ Yes
☐ No

I sent my kids to stay with friends or relatives
☐ Yes
☐ No

I made sure there were other people around
☐ Yes
☐ No

I fought back physically against my partner
☐ Yes
☐ No

I slept seperately from my partner
☐ Yes
☐ No

I refused to do what my partner wanted
☐ Yes
☐ No

I used or threatened to use a weapon against my partner
☐ Yes
☐ No

I left home to get away from my partner
☐ Yes
☐ No

I ended (or tried to end) the relationship with my partner
☐ Yes
☐ No

I fought back verbally against my partner
☐ Yes
☐ No

I tried to keep things quiet for my partner
☐ Yes
☐ No

I did whatever my partner wanted
☐ Yes
☐ No

I tried not to cry
☐ Yes
☐ No

I tried to avoid my partner
☐ Yes
☐ No

I tried to avoid an argument with my partner
☐ Yes
☐ No

I used alcohol or drugs
☐ Yes
☐ No

I exercised
☐ Yes
☐ No

I reached out to my friends or family for support
☐ Yes
☐ No

2 Can you think of anything else you have done to cope with relationship stress or problems in the past year? ________________________________

3 In the PAST YEAR, has a doctor, nurse, or other healthcare professional asked you or talked to you about domestic violence, or about concerns about safety or violence in your home? (Answer yes if you have been asked either in person, or on a form filled out at your clinic.)

- ☐ Yes
- ☐ No

C4. This section will ask about how you feel about yourself.

For each of the following statements, please indicate if you strongly agree, agree, disagree, or strongly disagree.

I feel that I am a person of worth, at least on an equal basis with others
☐ Strongly Agree
☐ Agree
☐ Disagree
☐ Strongly Disagree

I feel that I have a number of good qualities
☐ Strongly Agree
☐ Agree
☐ Disagree
☐ Strongly Disagree

All in all, I feel that I am failure
☐ Strongly Agree
☐ Agree
☐ Disagree
☐ Strongly Disagree

I am able to do things as well as most other people
☐ Strongly Agree
☐ Agree
☐ Disagree
☐ Strongly Disagree

I feel that I do not have much to be proud of
☐ Strongly Agree
☐ Agree
☐ Disagree
☐ Strongly Disagree

I take a positive attitude toward myself
☐ Strongly Agree
☐ Agree
☐ Disagree
☐ Strongly Disagree

On the whole, I am satisfied with myself
☐ Strongly Agree
☐ Agree
☐ Disagree
☐ Strongly Disagree

I wish I could have more respect for myself
☐ Strongly Agree
☐ Agree
☐ Disagree
☐ Strongly Disagree

I certainly feel useless at times
☐ Strongly Agree
☐ Agree
☐ Disagree
☐ Strongly Disagree

At times, I think I am no good at all
☐ Strongly Agree
☐ Agree
☐ Disagree
☐ Strongly Disagree

C5. Please help us understand how you think people in your community or neighborhood respond to family or domestic violence.

In my community or neighborhood, I think people would...

Try to break up a fight between a couple

- ☐ Strongly Agree
- ☐ Agree
- ☐ Neither Agree or Disagree
- ☐ Disagree
- ☐ Strongly Disagree

Try to convince a woman who is being abused that she should leave her partner

- ☐ Strongly Agree
- ☐ Agree
- ☐ Neither Agree or Disagree
- ☐ Disagree
- ☐ Strongly Disagree

Offer a woman who is being abused a place to stay

- ☐ Strongly Agree
- ☐ Agree
- ☐ Neither Agree or Disagree
- ☐ Disagree
- ☐ Strongly Disagree

Try to convince a woman who is being abused that she and her partner should get counseling

- ☐ Strongly Agree
- ☐ Agree
- ☐ Neither Agree or Disagree
- ☐ Disagree
- ☐ Strongly Disagree

Go out of their way to try to help a woman who is being abused

- ☐ Strongly Agree
- ☐ Agree
- ☐ Neither Agree or Disagree
- ☐ Disagree
- ☐ Strongly Disagree

Call the police when they hear or see a couple physically fighting

- ☐ Strongly Agree
- ☐ Agree
- ☐ Neither Agree or Disagree
- ☐ Disagree
- ☐ Strongly Disagree

Call the police when they hear or see a couple yelling and screaming

- ☐ Strongly Agree
- ☐ Agree
- ☐ Neither Agree or Disagree
- ☐ Disagree
- ☐ Strongly Disagree

I think that a woman who is being abused should feel comfortable talking to her neighbors about it

- ☐ Strongly Agree
- ☐ Agree
- ☐ Neither Agree or Disagree
- ☐ Disagree
- ☐ Strongly Disagree

C6. Sexual violence is common in women's lives and can affect women's health.

1 Has anyone ever exposed the sex organs of their body to you when you did not want it?
☐ Yes
☐ No

How old were you when this most recently occurred? ______________________

2 Has anyone ever threatened to have sex with you when you did not want it?
☐ Yes
☐ No

How old were you when this most recently occurred? ______________________

3 Has anyone ever touched the sex organs of your body when you did not want this?
☐ Yes
☐ No

How old were you when this most recently occurred? ______________________

4 Has anyone ever made you touch the sex organs of their body when you did not want this?
☐ Yes
☐ No

How old were you when this most recently occurred? ______________________

5 Has anyone ever forced you to have sex when you did not want this?
☐ Yes
☐ No

How old were you when this most recently occurred? ______________________

6 Have you had any other unwanted sexual experiences not mentioned above?
☐ Yes
☐ No

Please specify: ______________________

How old were you when this most recently occurred? ______________________

If you need to talk to someone regarding rape or sexual violence please contact the National Sexual Assault Hotline at 1-800-656-HOPE (4673), a free and confidential service. Information call also be found at the Rape, Abuse & Incest National Network website: http://www.rainn.org/.

Call 911 or your local emergency number if you are in immediate danger.

C7. Experiences during childhood can affect your health as an adult. While you were growing up, during your first 18 years of life:

1 Did a parent or other adult in the household often or very often... Swear at you, insult you, put you down, or humiliate you? or Act in a way that made you afraid that you might be physically hurt?
☐ Yes
☐ No

2 Did a parent or other adult in the household often or very often... Push, grab, slap, or throw something at you? or Ever hit you so hard that you had marks or were injured?
☐ Yes
☐ No

3 Did an adult or person at least 5 years older than you ever... Touch or fondle you or have you touch their body in a sexual way? or Attempt or actually have oral, anal, or vaginal intercourse with you?
☐ Yes
☐ No

4 Did you often or very often feel that ... No one in your family loved you or thought you were important or special? or Your family didn't look out for each other, feel close to each other, or support each other?
☐ Yes
☐ No

5 Did you often or very often feel that ... You didn't have enough to eat, had to wear dirty clothes, and had no one to protect you? or Your parents were too drunk or high to take care of you or take you to the doctor if you needed it?
☐ Yes
☐ No

6 Were your parents ever separated or divorced?
☐ Yes
☐ No

7 Was your mother or stepmother: Often or very often pushed, grabbed, slapped, or had something thrown at her? or Sometimes, often, or very often kicked, bitten, hit with a fist, or with something hard?
☐ Yes
☐ No

8 Did you live with anyone who was a problem drinker or alcoholic or who used street drugs?
☐ Yes
☐ No

9 Was a household member depressed or mentally ill, or did a household member attempt suicide?
☐ Yes
☐ No

10 Did a household member go to prison?
☐ Yes
☐ No

If you need support as a survivor of abuse, if you believe you are at risk of becoming an abuser, or if you know a child in danger, please call 1-800-4-A-CHILD (1-800-422-4453) to speak with a qualified crisis counselor or go online to http://www.childhelp.org for more information.

C8. We are interested in learning more about the support that is available to you.

1 About how many close friends and relatives do you have whom you feel at ease with and can talk to about what is on your mind.

____
(Number)

2 How often is each of the following kinds of support available to you if you need it?

Someone to take you to the doctor if you need it
- ☐ None of the time
- ☐ A little of the time
- ☐ Some of the time
- ☐ Most of the time
- ☐ All of the time

Someone who shows you love and affection
- ☐ None of the time
- ☐ A little of the time
- ☐ Some of the time
- ☐ Most of the time
- ☐ All of the time

Someone to confide in or talk to about yourself or your problems
- ☐ None of the time
- ☐ A little of the time
- ☐ Some of the time
- ☐ Most of the time
- ☐ All of the time

Someone to get together with for relaxation
- ☐ None of the time
- ☐ A little of the time
- ☐ Some of the time
- ☐ Most of the time
- ☐ All of the time

Someone to help with daily chores if you were sick
- ☐ None of the time
- ☐ A little of the time
- ☐ Some of the time
- ☐ Most of the time
- ☐ All of the time

Someone to share your private worries and fears with
- ☐ None of the time
- ☐ A little of the time
- ☐ Some of the time
- ☐ Most of the time
- ☐ All of the time

Someone to do something enjoyable with
- ☐ None of the time
- ☐ A little of the time
- ☐ Some of the time
- ☐ Most of the time
- ☐ All of the time

Someone to love and make you feel wanted
- ☐ None of the time
- ☐ A little of the time
- ☐ Some of the time
- ☐ Most of the time
- ☐ All of the time

D1. The next section asks you about how you get healthcare.

1 When you are sick or want medical advice, do you have a regular doctor or health care provider that you usually go to?

☐ Yes
☐ No

2 In the PAST YEAR, how many times have you visited a doctor or health care provider for any reason, not counting emergency room visits or overnight stays in a hospital?

____
(Number may be 0)

3 In the PAST YEAR, how many times have you visited an emergency room or stayed overnight in the hospital?

____

4 Thinking over the PAST YEAR, please let me know if any of the following has happened. You...

Had a health problem and needed to see a doctor but did not

☐ Yes
☐ No

Were not able to see a specialist when you thought you needed one

☐ Yes
☐ No

Felt you did not have enough personal control over decisions affecting your own medical care

☐ Yes
☐ No

Had concerns about the quality of care you received

☐ Yes
☐ No

Had difficulty getting care because there were not enough doctors or clinics near where you live

☐ Yes
☐ No

Tried to see a new doctor but could not because the doctor was not taking new patients

☐ Yes
☐ No

Did not fill prescription medication because of the cost

☐ Yes
☐ No

D2. Now thinking about the PAST YEAR, have you ever had to delay or not get the health care you needed because of any of the following:

You couldn't afford it	☐ Yes<br>☐ No
You didn't have health insurance	☐ Yes<br>☐ No
You had problems getting child care	☐ Yes<br>☐ No
You had transportation problems	☐ Yes<br>☐ No
You couldn't find time or you couldn't easily take time off work	☐ Yes<br>☐ No
You couldn't get an appointment with the doctor you wanted to see	☐ Yes<br>☐ No
Has anything else caused you to delay or prevented you from getting the health care you needed?	☐ Yes<br>☐ No
Please explain:	____________________

D3. This section will ask about your health insurance coverage.

1 Are you now covered by any form of health insurance or health plan, including any private insurance plan or a government program such as Medicare or Medicaid, or don't you have health insurance at this time?

- ☐ Yes, I have insurance
- ☐ No, I do not have insurance

2 Which of the following types of health insurance do you have? (Check all that apply)

- ☐ Private insurance through an employer, either your own or a family member's
- ☐ Private insurance you purchased directly from an insurance company such as an individual policy
- ☐ Medicaid, the government program that helps pay medical bills for people with low incomes
- ☐ Some other government medical program such as Medicare, CHAMPUS or the VA
- ☐ Other

Please describe: ______________________________

E1. The following questions will help us understand more about your background.

1 Describe your employment status, are you...? (Check all that apply)

- ☐ Employed full-time
- ☐ Employed part-time
- ☐ Retired
- ☐ Not employed for pay
- ☐ Disabled
- ☐ Student
- ☐ Self-employed
- ☐ Other

Other: ______________________________

2 Do you have a disability, handicap, or chronic disease that keeps you from participating fully in school, work, housework, or other activities?

- ☐ Yes
- ☐ No

3 Do you currently receive any of the following?

Food stamps

- ☐ Yes
- ☐ No

SSI-Supplemental Security Income

- ☐ Yes
- ☐ No

Public assistance or welfare payments from the state or local welfare office

- ☐ Yes
- ☐ No

Unemployment assistance

- ☐ Yes
- ☐ No

E2.

1 Was your family's total combined income before taxes LAST YEAR less than $20,000, or $20,000 or more?

☐ Less than $20,000
☐ $20,000 or more

Which of the following income best describes your family's total household income LAST YEAR before taxes, from all sources?

☐ Less than $1,000
☐ $1,000 to $1,999
☐ $2,000 to $2,999
☐ $3,000 to $3,999
☐ $4,000 to $4,999
☐ $5,000 to $5,999
☐ $6,000 to $6,999
☐ $7,000 to $7,999
☐ $8,000 to $8,999
☐ $9,000 to $9,999
☐ $10,000 to $10,999
☐ $11,000 to $11,999
☐ $12,000 to $12,999
☐ $13,000 to $13,999
☐ $14,000 to $14,999
☐ $15,000 to $15,999
☐ $16,000 to $16,999
☐ $17,000 to $17,999
☐ $18,000 to $18,999
☐ $19,000 to $19,999

Which of the following income categories best describes your total household income LAST YEAR?

☐ $20,000 to $20,999
☐ $21.000 to $21,999
☐ $22,000 to $22,999
☐ $23,000 to $23,999
☐ $24,000 to $24,999
☐ $25,000 to $25,999
☐ $26,000 to $26,999
☐ $27,000 to $27,999
☐ $28,000 to $28,999
☐ $29,000 to $29,999
☐ $30,000 to $30,999
☐ $31,000 to $31,999
☐ $32,000 to $32,999
☐ $33,000 to $33,999
☐ $34,000 to $34,999
☐ $35,000 to $39,999
☐ $40,000 to $44,999
☐ $45,000 to $49,999
☐ $50,000 to $54,999
☐ $55,000 to $59,999
☐ $60,000 to $64,999
☐ $65,000 to $69,999
☐ $70,000 to $74,999
☐ $75,000 and over

2 Counting yourself, how many adults 18 and older currently live in your household? ______________________

3 How many children under the age of 18 are currently living in your household? ______________________
