# Supplementary material for: Perceptions of firearms in a cohort of women exposed to intimate partner violence (IPV) in Central Pennsylvania
Source: BMC Womens Health. 2021 Jan 8;21:20. doi: 10.1186/s12905-020-01134-y (PMC7791958; doi:10.1186/s12905-020-01134-y)
Supplement: Supplementary file 3 — Additional file 3: One year follow up survey. [file 12905_2020_1134_MOESM3_ESM.pdf]

Women's Health Survey

Thank you for taking the time to complete this survey for the Women's Health Study at Penn State.

This survey will take approximately 30 minutes.

Remember, in this women's health survey, we ask some questions that can be personal. We ask that you are in a safe and private location and that you are confident that nobody can see your responses if you do not want them to.

You may skip any questions that you would prefer not to answer.

You may stop the survey, or save and resume, at any time.

There are no correct or incorrect responses.

Your completion of the survey implies your voluntary consent to participate in this research. If you would like to review your rights as a research participant, please review the consent document below.

[Attachment: "Summary Explanation of Research and Consent Form - Womens Health Survey.pdf"]

Please enter your Women's Health Survey ID Number: ______________________ (6 digits)

Please click the "now" button to the right, and begin the survey. ______________________

A1. The first few questions will help us understand your overall health.

1 In general, would you say your health is:

○ Excellent ○ Very good ○ Good ○ Fair ○ Poor

2 How tall are you without shoes?

○ shorter than 4' 5"
○ 4' 5"
○ 4' 6"
○ 4' 7"
○ 4', 8"
○ 4' 9"
○ 4' 10"
○ 4' 11"
○ 5' 0"
○ 5' 1"
○ 5' 2"
○ 5' 3"
○ 5' 4"
○ 5' 5"
○ 5' 6"
○ 5' 7"
○ 5' 8"
○ 5' 9"
○ 5' 10"
○ 5' 11"
○ 6' 0"
○ 6' 1"
○ 6' 2"
○ 6' 3"
○ 6' 4"
○ taller than 6' 4"

3 How much do you weigh without clothes or shoes? (If you are pregnant, how much did you weigh before your pregnancy?)

____
(Number of pounds (lbs))

We are interested in finding out about the kinds of physical activities that people do as part of their everyday lives. The next questions are about the time you spent being physically active during the last 7 days.

In answering the following questions:
-VIGOROUS - vigorous physical activities refer to activities that take hard physical effort and make you breathe much harder than normal.
-MODERATE - moderate activities refer to activities that take moderate physical effort and make you breathe somewhat harder than normal.

Please answer each question even if you do not consider yourself to be an active person.

4 During the last 7 days, did you do any vigorous physical activities like heavy lifting, digging, aerobics, or fast bicycling? Think about ONLY those physical activities that you did for at least 10 minutes at a time.

○ Yes
○ No

How many days in the past week did you do any vigorous physical activity?

____
(Number only)

How much time in total did you usually spend on one of those days doing vigorous physical activities?

◯ 30 minutes
◯ 1 hour
◯ 1.5 hours
◯ 2 hours
◯ 2.5 hours
◯ 3 hours
◯ 3.5 hours
◯ 4 hours
◯ 5 hours
◯ 6 hours
◯ 7 hours
◯ 8 hours +

5 During the last 7 days, did you do moderate physical activities like carrying light loads, bicycling at a regular pace, or doubles tennis? Again, think ONLY about those physical activities that you did for at least 10 minutes at a time.

◯ Yes
◯ No

How many days in the past week did you do any moderate physical activity?

____
(Number only)

How much time in total did you usually spend on one of those days doing moderate physical activities?

◯ 30 minutes
◯ 1 hour
◯ 1.5 hours
◯ 2 hours
◯ 2.5 hours
◯ 3 hours
◯ 3.5 hours
◯ 4 hours
◯ 5 hours
◯ 6 hours
◯ 7 hours
◯ 8 hours +

6 During the last 7 days, did you walk for at least 10 minutes at a time? This includes walking at work and at home, walking to travel from place to place, and any other walking that you did solely for recreation, sport, exercise, or leisure.

◯ Yes
◯ No

How many days in the past week did you walk for more than 10 minutes?

____
(Number only)

How much time in total did you usually spend walking on one of those days?

◯ 30 minutes
◯ 1 hour
◯ 1.5 hours
◯ 2 hours
◯ 2.5 hours
◯ 3 hours
◯ 3.5 hours
◯ 4 hours
◯ 5 hours
◯ 6 hours
◯ 7 hours
◯ 8 hours +

The last question is about the time you spent SITTING on the weekdays while at work, at home, while doing course work, and during leisure time. This includes time spent sitting at a desk, visiting friends, reading, traveling on a bus, or sitting or lying down to watch television.

7 During the last 7 days, how much time in total did you usually spend sitting on a week day?

◯ 30 minutes
◯ 1 hour
◯ 1.5 hours
◯ 2 hours
◯ 2.5 hours
◯ 3 hours
◯ 3.5 hours
◯ 4 hours
◯ 5 hours
◯ 6 hours
◯ 7 hours
◯ 8 hours +

A2. In the PAST YEAR, how many times have you used the following?

1 4 or more drinks of alcohol in one day

◯ Never ◯ Once or twice ◯ Monthly ◯ Weekly ◯ Daily or almost daily

2 Tobacco products, such as cigarettes, cigars, or chewing tobacco

◯ Never ◯ Once or twice ◯ Monthly ◯ Weekly ◯ Daily or almost daily

3 Prescription drugs for NONmedical reasons (for example: taking a pain medication to relax instead of for pain relief)

◯ Never ◯ Once or twice ◯ Monthly ◯ Weekly ◯ Daily or almost daily

4 Illegal drugs

◯ Never ◯ Once or twice ◯ Monthly ◯ Weekly ◯ Daily or almost daily

Some women struggle with drug or alcohol issues. If you would like more information about these issues call the National Helpline at 800-622-HELP (4357) or visit the online treatment locator at http://www.samhsa.gov/treatment.

A3. This section will ask about your eating habits.

1	Are you satisfied with your eating patterns?	◯ Yes<br>◯ No
2	Do you ever eat in secret?	◯ Yes<br>◯ No
3	Does your weight affect the way you feel about yourself?	◯ Yes<br>◯ No
4	Have any members of your family suffered with an eating disorder?	◯ Yes<br>◯ No
5	Do you currently suffer with or have you ever suffered in the past with an eating disorder?	◯ Yes<br>◯ No

If you are concerned that you or someone you care about is affected by an eating disorder, you may find out more information on the National Eating Disorders Association website: http://www.nationaleatingdisorders.org/. A toll free, confidential helpline is available Monday-Friday, 9:00 am- 5:00 pm, Eastern Standard Time: 1-800-931-2237.

B. In the next section, we are interested in understanding more about your mood and feelings.

1 Over the last TWO WEEKS, how often have you felt any of the following?

I feel sad, down in the dumps, or unhappy

◯ Not at all ◯ Rarely ◯ Sometimes ◯ Often ◯ Most of the time

I can't concentrate or focus

◯ Not at all ◯ Rarely ◯ Sometimes ◯ Often ◯ Most of the time

Nothing seems to give me much pleasure

◯ Not at all ◯ Rarely ◯ Sometimes ◯ Often ◯ Most of the time

I feel tired, I have no energy

◯ Not at all ◯ Rarely ◯ Sometimes ◯ Often ◯ Most of the time

I have had thoughts of suicide

◯ Not at all ◯ Rarely ◯ Sometimes ◯ Often ◯ Most of the time

If you are thinking of harming yourself, please call 911, your local emergency number, or the National Suicide Prevention Hotline at: 800-273-TALK (8255).

Please check here to indicate that you understand that you should seek help if you are feeling suicidal. ☐ I understand

2 Over the last TWO WEEKS, how often have you felt any of the following?

I have difficulty sleeping

◯ Not at all ◯ Rarely ◯ Sometimes ◯ Often ◯ Most of the time

I have been sleeping too much

◯ Not at all ◯ Rarely ◯ Sometimes ◯ Often ◯ Most of the time

I have lost my appetite

◯ Not at all ◯ Rarely ◯ Sometimes ◯ Often ◯ Most of the time

I have been eating more

◯ Not at all ◯ Rarely ◯ Sometimes ◯ Often ◯ Most of the time

I feel tense, anxious, or can't sit still

◯ Not at all ◯ Rarely ◯ Sometimes ◯ Often ◯ Most of the time

I feel worried or fearful

◯ Not at all ◯ Rarely ◯ Sometimes ◯ Often ◯ Most of the time

I have attacks of anxiety or panic

◯ Not at all ◯ Rarely ◯ Sometimes ◯ Often ◯ Most of the time

I worry about dying or losing control

◯ Not at all ◯ Rarely ◯ Sometimes ◯ Often ◯ Most of the time

I am nervous or shaky in social situations

◯ Not at all ◯ Rarely ◯ Sometimes ◯ Often ◯ Most of the time

I have nightmares or flashbacks

◯ Not at all ◯ Rarely ◯ Sometimes ◯ Often ◯ Most of the time

I am jumpy or feel startled easily

◯ Not at all ◯ Rarely ◯ Sometimes ◯ Often ◯ Most of the time

I avoid places that strongly remind me of a bad experience

◯ Not at all ◯ Rarely ◯ Sometimes ◯ Often ◯ Most of the time

I feel dull, numb, or detached

◯ Not at all ◯ Rarely ◯ Sometimes ◯ Often ◯ Most of the time

I can't get certain thoughts out of my mind

◯ Not at all ◯ Rarely ◯ Sometimes ◯ Often ◯ Most of the time

I feel I must repeat certain acts or rituals

◯ Not at all ◯ Rarely ◯ Sometimes ◯ Often ◯ Most of the time

I feel the need to check and recheck things

◯ Not at all ◯ Rarely ◯ Sometimes ◯ Often ◯ Most of the time

3a AT ANY TIME IN YOUR LIFE have you:

Had more energy than usual

◯ Not at all ◯ Rarely ◯ Sometimes ◯ Often ◯ Most of the time

Felt unusually irritable or angry

◯ Not at all ◯ Rarely ◯ Sometimes ◯ Often ◯ Most of the time

Felt unusually excited, revved up, or high

◯ Not at all ◯ Rarely ◯ Sometimes ◯ Often ◯ Most of the time

Needed less sleep than usual

◯ Not at all ◯ Rarely ◯ Sometimes ◯ Often ◯ Most of the time

3b Please indicate whether any of the above symptoms:

Interfere with work or school

◯ Not at all ◯ Rarely ◯ Sometimes ◯ Often ◯ Most of the time

Affect my relationships with friends or family

◯ Not at all ◯ Rarely ◯ Sometimes ◯ Often ◯ Most of the time

Have led to my using alcohol to get by

◯ Not at all ◯ Rarely ◯ Sometimes ◯ Often ◯ Most of the time

Have led to my using illegal drugs

◯ Not at all ◯ Rarely ◯ Sometimes ◯ Often ◯ Most of the time

Some women struggle with drug, alcohol or other mental health issues. If you would like more information about these issues call the National Helpline at 800-662-HELP (4357) or visit the online treatment locator at http://www.samhsa.gov/treatment.

If you are thinking of harming yourself, please call 911, your local emergency number, or the National Suicide Prevention Hotline at: 800-273-TALK (8255).

4 Are you currently receiving treatment from a doctor or other health professional for any type of mental health condition or emotional problem?

◯ Yes
◯ No

5 In your LIFETIME, have you received treatment from a doctor or other health professional for any type of mental health condition or emotional problem?

◯ Yes
◯ No

C1. The next section will ask you questions about your interpersonal relationships.

1 Have you been in a relationship with a boyfriend, girlfriend, husband, wife, or other partner in the PAST YEAR?

- ◯ Yes
- ◯ No

2 Do you consider yourself to be:

- ◯ Heterosexual or straight
- ◯ Gay or lesbian
- ◯ Bisexual
- ◯ Other

Other: ______________________________

3 In the PAST YEAR, who have you had sex with?

- ◯ Men only
- ◯ Women only
- ◯ Both men and women
- ◯ I did not have sex in the past year
- ◯ Other

Other: ______________________________

4 In your lifetime, who have you had sex with?

- ◯ Men only
- ◯ Women only
- ◯ Both men and women
- ◯ I have never had sex
- ◯ Other

Other: ______________________________

C2. Sexual violence is common in women's lives and can affect women's health. The next questions are about sexual violence you may have experienced, by people you know or don't know.

1 In the PAST YEAR, has anyone exposed their sex organs to you when you did not want it?
◯ Yes
◯ No

2 In the PAST YEAR, has anyone threatened to have sex with you when you did not want it?
◯ Yes
◯ No

3 In the PAST YEAR, has anyone touched your sex organs when you did not want it?
◯ Yes
◯ No

4 In the PAST YEAR, has anyone made you touch their sex organs when you did not want it?
◯ Yes
◯ No

5 In the PAST YEAR, has anyone ever forced you to have sex when you did not want it?
◯ Yes
◯ No

6 In the PAST YEAR, have you had any other unwanted sexual experiences not mentioned above?
◯ Yes
◯ No

Please specify: ______________________________

If you answered "Yes" to any of the above questions, you may have experienced rape or sexual violence.

If you need to talk to someone regarding rape or sexual violence please contact the National Sexual Assault Hotline at 1-800-656-HOPE (4673), a free and confidential service. Information call also be found at the Rape, Abuse & Incest National Network website: http://www.rainn.org/.

Call 911 or your local emergency number if you are in immediate danger.

C3. Relationship stress and violence are common in many people's lives and can affect women's health.

1 In the past year, have you been humiliated or emotionally abused in other ways by your partner or ex-partner?
◯ Yes
◯ No

Has this become more frequent over the PAST YEAR?
◯ Yes
◯ No

Has this become more severe over the PAST YEAR?
◯ Yes
◯ No

2 In the past year, have you been afraid of your partner or ex-partner?
◯ Yes
◯ No

Has this become more frequent over the PAST YEAR?
◯ Yes
◯ No

Has this become more severe over the PAST YEAR?
◯ Yes
◯ No

3 In the past year, have you been raped or forced to have any kind of sexual activity by your partner or ex-partner?
◯ Yes
◯ No

Has this become more frequent over the past year?
◯ Yes
◯ No

Has this become more severe over the PAST YEAR?
◯ Yes
◯ No

4 In the past year, have you been kicked, hit, slapped, or otherwise physically hurt by your partner or ex-partner?
◯ Yes
◯ No

Has this become more frequent over the PAST YEAR?
◯ Yes
◯ No

Has this become more severe over the PAST YEAR?
◯ Yes
◯ No

5 Has a partner or ex-partner ever messed with your birth control or tried to get you pregnant when you didn't want to be?
◯ Yes
◯ No

Has this happened within the past year?
◯ Yes
◯ No

6 Has a partner or ex-partner ever refused to use condoms when you asked?
◯ Yes
◯ No

Has this happened in the past year?
◯ Yes
◯ No

7 During the past year, how many times has a partner prevented you from going to a clinic or seeing a doctor or nurse when you wanted to?
____
(Number only)

8 During the past year, how often has a partner tried to interfere with your healthcare?
◯ Never
◯ Once or twice
◯ A few times
◯ Very often

If you need to talk about domestic violence or abuse feel free to call this toll-free and confidential hotline 1-800-799-SAFE (7233). Information can also be found at: http://www.thehotline.org.

If you need to talk to someone regarding rape or sexual violence please contact the National Sexual Assault Hotline at 1-800-656-HOPE (4673), a free and confidential service. Information can also be found at the Rape, Abuse & Incest National Network website: http://www.rainn.org/.

Call 911 or your local emergency number if you are in immediate danger.

C4. Women who experience problems in their relationships have many ways of coping or seeking help.

1 Have you done any of the following because of a problem with your partner or ex-partner?

I tried to get help from a member of the clergy
- ◯ Yes, within the last year
- ◯ Yes, in my adult lifetime but not within the last year
- ◯ No

I tried to get help from an employer or coworker
- ◯ Yes, within the last year
- ◯ Yes, in my adult lifetime but not within the last year
- ◯ No

I talked to a doctor or nurse about abuse or relationship trouble
- ◯ Yes, within the last year
- ◯ Yes, in my adult lifetime but not within the last year
- ◯ No

I called a mental health counselor for myself
- ◯ Yes, within the last year
- ◯ Yes, in my adult lifetime but not within the last year
- ◯ No

I tried to get my partner into counseling
- ◯ Yes, within the last year
- ◯ Yes, in my adult lifetime but not within the last year
- ◯ No

I stayed in a shelter
- ◯ Yes, within the last year
- ◯ Yes, in my adult lifetime but not within the last year
- ◯ No

I talked to someone at a domestic violence program, shelter or hotline
- ◯ Yes, within the last year
- ◯ Yes, in my adult lifetime but not within the last year
- ◯ No

I tried to get help for alcohol or substance abuse
- ◯ Yes, within the last year
- ◯ Yes, in my adult lifetime but not within the last year
- ◯ No

I tried to get my partner help for alcohol or substance abuse
- ◯ Yes, within the last year
- ◯ Yes, in my adult lifetime but not within the last year
- ◯ No

I filed or tried to file for a protection order (or PFA)
- ◯ Yes, within the last year
- ◯ Yes, in my adult lifetime but not within the last year
- ◯ No

I filed or tried to file criminal charges
- ◯ Yes, within the last year
- ◯ Yes, in my adult lifetime but not within the last year
- ◯ No

I sought legal help or help from an attorney
- ◯ Yes, within the last year
- ◯ Yes, in my adult lifetime but not within the last year
- ◯ No

I called the police	◯ Yes, within the last year<br>◯ Yes, in my adult lifetime but not within the last year<br>◯ No
I hid the car or house keys	◯ Yes, within the last year<br>◯ Yes, in my adult lifetime but not within the last year<br>◯ No
I kept money and other valuables hidden	◯ Yes, within the last year<br>◯ Yes, in my adult lifetime but not within the last year<br>◯ No
I developed a code so others would know I was in danger	◯ Yes, within the last year<br>◯ Yes, in my adult lifetime but not within the last year<br>◯ No
I worked out an escape plan	◯ Yes, within the last year<br>◯ Yes, in my adult lifetime but not within the last year<br>◯ No
I removed or hid weapons	◯ Yes, within the last year<br>◯ Yes, in my adult lifetime but not within the last year<br>◯ No
I kept important phone numbers I could use to get help	◯ Yes, within the last year<br>◯ Yes, in my adult lifetime but not within the last year<br>◯ No
I kept an extra supply of basic necessities for myself or my children	◯ Yes, within the last year<br>◯ Yes, in my adult lifetime but not within the last year<br>◯ No
I hid important papers from my partner	◯ Yes, within the last year<br>◯ Yes, in my adult lifetime but not within the last year<br>◯ No
I put a knife, gun, or other weapon where I could get it	◯ Yes, within the last year<br>◯ Yes, in my adult lifetime but not within the last year<br>◯ No
I changed the locks or somehow improved security	◯ Yes, within the last year<br>◯ Yes, in my adult lifetime but not within the last year<br>◯ No
I talked to family or friends about what to do to protect me or my children	◯ Yes, within the last year<br>◯ Yes, in my adult lifetime but not within the last year<br>◯ No
I stayed with family or friends	◯ Yes, within the last year<br>◯ Yes, in my adult lifetime but not within the last year<br>◯ No

I sent my kids to stay with friends or relatives	◯ Yes, within the last year<br>◯ Yes, in my adult lifetime but not within the last year<br>◯ No
I made sure there were other people around	◯ Yes, within the last year<br>◯ Yes, in my adult lifetime but not within the last year<br>◯ No
I fought back physically against my partner	◯ Yes, within the last year<br>◯ Yes, in my adult lifetime but not within the last year<br>◯ No
I slept separately from my partner	◯ Yes, within the last year<br>◯ Yes, in my adult lifetime but not within the last year<br>◯ No
I refused to do what my partner wanted	◯ Yes, within the last year<br>◯ Yes, in my adult lifetime but not within the last year<br>◯ No
I used or threatened to use a weapon against my partner	◯ Yes, within the last year<br>◯ Yes, in my adult lifetime but not within the last year<br>◯ No
I left home to get away from my partner	◯ Yes, within the last year<br>◯ Yes, in my adult lifetime but not within the last year<br>◯ No
I ended (or tried to end) the relationship with my partner	◯ Yes, within the last year<br>◯ Yes, in my adult lifetime but not within the last year<br>◯ No
I fought back verbally against my partner	◯ Yes, within the last year<br>◯ Yes, in my adult lifetime but not within the last year<br>◯ No
I tried to keep things quiet for my partner	◯ Yes, within the last year<br>◯ Yes, in my adult lifetime but not within the last year<br>◯ No
I did whatever my partner wanted	◯ Yes, within the last year<br>◯ Yes, in my adult lifetime but not within the last year<br>◯ No
I tried not to cry	◯ Yes, within the last year<br>◯ Yes, in my adult lifetime but not within the last year<br>◯ No
I tried to avoid my partner	◯ Yes, within the last year<br>◯ Yes, in my adult lifetime but not within the last year<br>◯ No

I tried to avoid an argument with my partner	◯ Yes, within the last year<br>◯ Yes, in my adult lifetime but not within the last year<br>◯ No
I used alcohol or drugs	◯ Yes, within the last year<br>◯ Yes, in my adult lifetime but not within the last year<br>◯ No
I exercised to take my mind off things or make me feel better	◯ Yes, within the last year<br>◯ Yes, in my adult lifetime but not within the last year<br>◯ No
I reached out to my friends or family for support	◯ Yes, within the last year<br>◯ Yes, in my adult lifetime but not within the last year<br>◯ No
I tried to find comfort in my religion or prayed	◯ Yes, within the last year<br>◯ Yes, in my adult lifetime but not within the last year<br>◯ No
I talked things through or reasoned with my partner	◯ Yes, within the last year<br>◯ Yes, in my adult lifetime but not within the last year<br>◯ No
I did things for myself that make me feel good (for example, reading, writing, listening to music, or art)	◯ Yes, within the last year<br>◯ Yes, in my adult lifetime but not within the last year<br>◯ No
What did you do for yourself that made you feel good?	__________________________________
I did yoga or meditated	◯ Yes, within the last year<br>◯ Yes, in my adult lifetime but not within the last year<br>◯ No
I kept busy or distracted myself to take my mind off of things	◯ Yes, within the last year<br>◯ Yes, in my adult lifetime but not within the last year<br>◯ No
I went to a support group or meeting	◯ Yes, within the last year<br>◯ Yes, in my adult lifetime but not within the last year<br>◯ No
I ate more than usual	◯ Yes, within the last year<br>◯ Yes, in my adult lifetime but not within the last year<br>◯ No
I slept more than usual	◯ Yes, within the last year<br>◯ Yes, in my adult lifetime but not within the last year<br>◯ No
I turned to work to take my mind off of things	◯ Yes, within the last year<br>◯ Yes, in my adult lifetime but not within the last year<br>◯ No

I talked with a health care professional about related symptoms, but not about the relationship problems	◯ Yes, within the last year<br>◯ Yes, in my adult lifetime but not within the last year<br>◯ No
I took prescription drugs	◯ Yes, within the last year<br>◯ Yes, in my adult lifetime but not within the last year<br>◯ No
I hid things from my partner	◯ Yes, within the last year<br>◯ Yes, in my adult lifetime but not within the last year<br>◯ No
I kept information from my partner	◯ Yes, within the last year<br>◯ Yes, in my adult lifetime but not within the last year<br>◯ No
I tried to get help from the court/legal system	◯ Yes, within the last year<br>◯ Yes, in my adult lifetime but not within the last year<br>◯ No
I tried to get my partner medical help or counseling	◯ Yes, within the last year<br>◯ Yes, in my adult lifetime but not within the last year<br>◯ No
I got upset and let my emotions out or cried	◯ Yes, within the last year<br>◯ Yes, in my adult lifetime but not within the last year<br>◯ No

2 Can you think of anything else you have done to cope with relationship stress or problems?

C5. What do you think people who live near you would do if they saw or heard domestic violence?

1 People who live near me...

Would go out of their way to try to help a woman who is being abused

◯ Strongly Disagree ◯ Disagree ◯ Agree ◯ Strongly Agree

Would try to convince a woman who is being abused that she and her partner should get counseling

◯ Strongly Disagree ◯ Disagree ◯ Agree ◯ Strongly Agree

Would not try to break up a fight between a couple

◯ Strongly Disagree ◯ Disagree ◯ Agree ◯ Strongly Agree

Would try to convince a woman who is being abused that she should leave her partner

◯ Strongly Disagree ◯ Disagree ◯ Agree ◯ Strongly Agree

Would not call the police when they hear or see a couple yelling and screaming

◯ Strongly Disagree ◯ Disagree ◯ Agree ◯ Strongly Agree

Would call the police when they hear or see a couple physically fighting

◯ Strongly Disagree ◯ Disagree ◯ Agree ◯ Strongly Agree

Would offer a woman who is being abused a place to stay

◯ Strongly Disagree ◯ Disagree ◯ Agree ◯ Strongly Agree

What do you think people who live near you think about domestic violence?

2 People who live near me...

Think that couples' fighting is just a normal part of being together

◯ Strongly Disagree ◯ Disagree ◯ Agree ◯ Stronly Agree

Don't know how to help a woman who is being abused

◯ Strongly Disagree ◯ Disagree ◯ Agree ◯ Strongly Agree

Believe that there is no excuse for a partner to beat up on a woman

◯ Strongly Disagree ◯ Disagree ◯ Agree ◯ Strongly Agree

Believe that couples' fighting is a private matter

◯ Strongly Disagree ◯ Disagree ◯ Agree ◯ Strongly Agree

Think that domestic violence is not a problem in our community

◯ Strongly Disagree ◯ Disagree ◯ Agree ◯ Strongly Agree

Think it's a woman's own fault if she is being abused

◯ Strongly Disagree ◯ Disagree ◯ Agree ◯ Strongly Agree

Generally know very little about domestic violence

◯ Strongly Disagree ◯ Disagree ◯ Agree ◯ Strongly Agree

Fear for their own safety when deciding to help a woman who is being abused

◯ Strongly Disagree ◯ Disagree ◯ Agree ◯ Strongly Agree

C6. This next section asks you about guns in your community.

1 How easy is it for people who live near you to get a gun?

◯ Very Easy ◯ Easy ◯ Hard ◯ Impossible

2 Are any firearms kept in or around your home? (Include those kept in a garage, outdoor storage area, car, truck or other motor vehicle.)

◯ Yes
◯ No

3 Does having a gun around make you feel safer or less safe?

◯ Very safe ◯ Somewhat safer ◯ Somewhat less safe ◯ Very unsafe

4 Please indicate how much you agree or disagree with the following statement: People who live near me would use a gun to protect a woman who is being abused.

◯ Strongly Disagree ◯ Disagree ◯ Agree ◯ Strongly Agree

C7. We are interested in learning more about the support that is available to you.

1 About how many close friends and relatives do you have whom you feel at ease with and can talk to about what is on your mind? ______________________ (Number)

2 How often is each of the following kinds of support available to you if you need it?

Someone to take you to the doctor if you need it

◯ None of the time ◯ A little of the time ◯ Some of the time ◯ Most of the time ◯ All of the time

Someone who shows you love and affection

◯ None of the time ◯ A little of the time ◯ Some of the time ◯ Most of the time ◯ All of the time

Someone to confide in or talk to about yourself or your problems

◯ None of the time ◯ A little of the time ◯ Some of the time ◯ Most of the time ◯ All of the time

Someone to get together with for relaxation

◯ None of the time ◯ A little of the time ◯ Some of the time ◯ Most of the time ◯ All of the time

Someone to help with daily chores if you were sick

◯ None of the time ◯ A little of the time ◯ Some of the time ◯ Most of the time ◯ All of the time

Someone to share your private worries and fears with

◯ None of the time ◯ A little of the time ◯ Some of the time ◯ Most of the time ◯ All of the time

Someone to do something enjoyable with

◯ None of the time ◯ A little of the time ◯ Some of the time ◯ Most of the time ◯ All of the time

Someone to love and make you feel wanted

◯ None of the time ◯ A little of the time ◯ Some of the time ◯ Most of the time ◯ All of the time

D1. The next section asks you about how you get healthcare.

1 When you are sick or want medical advice, do you have a regular doctor or health care provider that you usually go to?

◯ Yes
◯ No

2 In the past year, have you visited a doctor or other healthcare provider for any reason?

◯ Yes
◯ No

In the PAST YEAR, how many times have you visited a doctor or health care provider for any reason, not counting emergency room visits or overnight stays in a hospital?

____
(Number may be 0)

In the PAST YEAR, how many times have you visited an emergency room or stayed overnight in the hospital?

____
(Number may be 0)

D2. The next section asks about conversations you may have had with a healthcare provider within the past year.

In the PAST YEAR, has a doctor or other healthcare provider asked you about domestic violence or feeling unsafe at home?

- ◯ Yes
- ◯ No

Some women have conversations with their healthcare providers about domestic violence even if the healthcare provider does not ask them about it. In the PAST YEAR, did you ever talk to or have a conversation with a doctor or other healthcare provider about domestic violence or feeling unsafe at home?

- ◯ Yes
- ◯ No

Would you have liked to talk to a healthcare provider about domestic violence or whether you feel safe at home?

- ◯ Yes
- ◯ No

In the past year, did you tell a doctor or other healthcare provider that you were concerned about domestic violence or that you felt unsafe at home?

- ◯ Yes
- ◯ No

Thinking back to the LAST TIME you talked with a healthcare provider about domestic violence or feeling unsafe at home: Who brought it up?

- ◯ I brought it up
- ◯ My healthcare provider brought it up
- ◯ Other

Other (please specify): __________________________________

Thinking back to the LAST TIME you talked with a healthcare provider about domestic violence or feeling unsafe at home: What type of doctor or healthcare provider did you talk with?

- ◯ Primary Care Physician
- ◯ Nurse Practicioner or Physician Assistant
- ◯ Obstetrician/Gynecologist (OB/GYN)
- ◯ Psychiatrist, Psychologist, or Addiction Specialist
- ◯ Pediatrician
- ◯ Emergency Medicine or Urgent Care Provider
- ◯ Surgeon
- ◯ Other
- ◯ Don't Know

If other, please specify: __________________________________

Thinking back to the LAST TIME you talked with a healthcare provider about domestic violence or feeling unsafe at home: Was your healthcare provider male or female?

- ◯ Male
- ◯ Female

Thinking back to the LAST TIME you talked with a healthcare provider about domestic violence or feeling unsafe at home: Was there anybody else in the room during your discussion?

- ◯ Yes
- ◯ No

Who else was in the room?

- ☐ A nurse
- ☐ My spouse or partner
- ☐ Another family member
- ☐ My friend
- ☐ A medical or nursing student
- ☐ Other

(Check all that apply)

If other, please specify: __________________________________

Thinking back to the LAST TIME you talked with a healthcare provider about domestic violence or feeling unsafe at home: Which of the following things did you talk about?

- ☐ What was happening to me
- ☐ If I was worried
- ☐ My physical safety
- ☐ The safety of my children
- ☐ If my health was affected
- ☐ If my mood was affected
- ☐ If my relationships with my friends or family were affected
- ☐ If my partner had a problem with alcohol or drugs
- ☐ Other

(Check all that apply)

If other, please specify: ______________________________

Thinking back to the LAST TIME you talked with a healthcare provider about domestic violence or feeling unsafe at home: What did you learn from the discussion?

- ☐ I learned I was not alone
- ☐ I learned how I could stay safe
- ☐ I learned where or how I could get help
- ☐ I learned about medications that could help me
- ☐ None of the above
- ☐ Other

(Check all that apply)

If other, please specify: ______________________________

Thinking back to the LAST TIME you talked with a healthcare provider about domestic violence or feeling unsafe at home: Which of the following things did you or the healthcare provider do after your discussion?

- ☐ I scheduled a follow-up appointment with my healthcare provider
- ☐ I received a prescription for medication
- ☐ I scheduled an appointment with a therapist or mental health provider
- ☐ My healthcare provider gave me information about domestic violence
- ☐ My healthcare provider gave me contact information for a social worker, domestic violence shelter, or other service
- ☐ My healthcare provider or I called the police
- ☐ None of the above
- ☐ Other

(Check all that apply)

If other, please specify: ______________________________

Thinking back to the LAST TIME you talked with a healthcare provider about domestic violence or feeling unsafe at home: Were you ever worried that what you told your healthcare provider would NOT be kept private?

- ◯ Yes
- ◯ No

Thinking back to the LAST TIME you talked with a healthcare provider about domestic violence or feeling unsafe at home: Was there anything that you wanted to talk to your healthcare provider about that you were not able to?

- ◯ Yes
- ◯ No

What would you have liked to talk about that you were not able to? ______________________________

Why do you feel you were unable to discuss this concern with your healthcare provider? ______________________________

Thinking back to the LAST TIME you discussed concerns about domestic violence or your safety at home with your healthcare provider:

How satisfied were you with the talk you had with your healthcare provider?

- ◯ Extremely satisfied
- ◯ Very satisfied
- ◯ Somewhat satisfied
- ◯ Somewhat dissatisfied
- ◯ Very dissatisfied

How comfortable were you discussing domestic violence or feeling unsafe at home with your healthcare provider?

- ◯ Extremely comfortable
- ◯ Very comfortable
- ◯ Somewhat comfortable
- ◯ Somewhat uncomfortable
- ◯ Very uncomfortable

Is there anything else you would like to tell us about the LAST TIME you talked with your healthcare provider about domestic violence or feeling unsafe at home?

____

E1. The following questions will help us to understand more about your background.

1 Are you now covered by any form of health insurance or health plan, including any private insurance plan or a government program such as Medicare or Medicaid, or don't you have health insurance at this time?

- ◯ Yes, I have insurance
- ◯ No, I do not have insurance

Which of the following types of health insurance do you have? (Check all that apply)

- ☐ Private insurance through an employer, either your own or a family member's
- ☐ Private insurance you purchased directly from an insurance company such as an individual policy
- ☐ Medicaid, the government program that helps pay medical bills for people with low incomes
- ☐ Some other government medical program such as Medicare, CHAMPUS or the VA
- ☐ Other

Please describe: __________________________________

2 Describe your employment status, are you...? (Check all that apply)

- ☐ Employed full-time
- ☐ Employed part-time
- ☐ Retired
- ☐ Not employed for pay
- ☐ Disabled
- ☐ Student
- ☐ Self-employed
- ☐ Other

Other: __________________________________

3 Was your family's total combined income before taxes LAST YEAR less than $20,000, or $20,000 or more?

- ◯ Less than $20,000
- ◯ $20,000 or more

Which of the following income best describes your family's total household income LAST YEAR before taxes, from all sources?

- ◯ Less than $1,000
- ◯ $1,000 to $1,999
- ◯ $2,000 to $2,999
- ◯ $3,000 to $3,999
- ◯ $4,000 to $4,999
- ◯ $5,000 to $5,999
- ◯ $6,000 to $6,999
- ◯ $7,000 to $7,999
- ◯ $8,000 to $8,999
- ◯ $9,000 to $9,999
- ◯ $10,000 to $10,999
- ◯ $11,000 to $11,999
- ◯ $12,000 to $12,999
- ◯ $13,000 to $13,999
- ◯ $14,000 to $14,999
- ◯ $15,000 to $15,999
- ◯ $16,000 to $16,999
- ◯ $17,000 to $17,999
- ◯ $18,000 to $18,999
- ◯ $19,000 to $19,999

	Which of the following income best describes your family's total household income LAST YEAR before taxes, from all sources?	◯ $20,000 to $20,999<br>◯ $21.000 to $21,999<br>◯ $22,000 to $22,999<br>◯ $23,000 to $23,999<br>◯ $24,000 to $24,999<br>◯ $25,000 to $25,999<br>◯ $26,000 to $26,999<br>◯ $27,000 to $27,999<br>◯ $28,000 to $28,999<br>◯ $29,000 to $29,999<br>◯ $30,000 to $30,999<br>◯ $31,000 to $31,999<br>◯ $32,000 to $32,999<br>◯ $33,000 to $33,999<br>◯ $34,000 to $34,999<br>◯ $35,000 to $39,999<br>◯ $40,000 to $44,999<br>◯ $45,000 to $49,999<br>◯ $50,000 to $54,999<br>◯ $55,000 to $59,999<br>◯ $60,000 to $64,999<br>◯ $65,000 to $69,999<br>◯ $70,000 to $74,999<br>◯ $75,000 and over
4	Have you or your child received benefits from WIC, the Special Supplemental Nutrition Program for Women, Infants, and Children?	◯ Yes, in the last 12 months<br>◯ Yes, in my lifetime<br>◯ No
5	Counting yourself, how many adults 18 or older currently live in your household?	______________________
6	Do you have any children under the age of 18 currently living in your household?	◯ Yes<br>◯ No
	How many children under the age of 18 are currently living in your household?	______________________
	How old is your first child?	______________________<br>(Only list children under 18.)
	Do you have another child to list?	◯ Yes<br>◯ No
	How old is your second child?	______________________<br>(Only list children under 18.)
	Do you have another child to list?	◯ Yes<br>◯ No
	How old is your third child?	______________________<br>(Only list children under 18.)
	Do you have another child to list?	◯ Yes<br>◯ No
	How old is your fourth child?	______________________<br>(Only list children under 18.)
	Do you have another child to list?	◯ Yes<br>◯ No
	How old is your fifth child under the age of 18?	______________________
	If you have more than five children who are under 18, please list the ages of any not listed above:	______________________

E2. Thank you! You are near the end of the survey.

Is there anything else you would like us to know that has not been covered? __________________________________
